# Breaking HER limits with Ni@B_40_’s single-atom catalytic prowess

**DOI:** 10.1038/s41598-026-46437-6

**Published:** 2026-03-31

**Authors:** Naveen Kosar, Saira Rafiq, Sumayya M. Ansari, Mona A. Aziz Aljar, Muhammad Imran, Ahmad Hasan, Imran Malik, Tariq Mahmood, Adnan Younis

**Affiliations:** 1 Chemistry Department, King Fahd University of Petroleum & Minerals, Dhahran, 31261 Saudi Arabia; 2 IRC-RAC, King Fahd University of Petroleum & Minerals, Dhahran, 31261 Saudi Arabia; 3 Department of Chemistry, University of Management and Technology (UMT), C-11, Johar Town, Lahore, Pakistan; 4 Department of Physics, United Arab Emirates University, P.O. Box 15551, Al Ain, United Arab Emirates; 5 Department of Chemistry, College of Science, University of Bahrain, Sakhir, 32038 Bahrain; 6 Research Center for Advanced Materials Science (RCAMS), Chemistry Department, Faculty of Science, King Khalid University, P.O. Box 9004, Abha, 61413 Saudi Arabia; 7 Department of Basic Sciences, Deanship of Preparatory Year and Supporting Studies, Imam Abdulrahman Bin Faisal University, P.O. Box 1982, Dammam, 34212 Saudi Arabia; 8 Department of Chemistry, COMSATS University Islamabad, Abbottabad Campus, Abbottabad, 22060 Pakistan

**Keywords:** B_40_, SAC, HER, DFT, Sustainability, Chemistry, Energy science and technology, Materials science

## Abstract

The hydrogen evolution reaction (HER) has emerged as a key process in the pursuit of sustainable alternatives to nonrenewable fuels. Single-atom catalysts (SACs) are particularly promising for HER electrocatalysis due to their exceptional atom utilization, high electrical conductivity, and thermal stability. In this study, we systematically evaluated the catalytic potential of late first-row transition metal-decorated TM@B_40_ (TM = Zn, Fe, Co, Cu, and Ni) complexes as SACs for HER using density functional theory (DFT) and ab initio molecular dynamic (AIMD) calculations. The interaction energies (E_int_ of these complexes ranged from − 1.16 to -3.72 eV at B3LYP-D3/6–31 + G (d) method in aqueous phase, confirming their thermodynamic stability. Notably, Ni@B_40_ and Cu@B_40_ exhibited the lowest Gibb’s free energy of -0.01 eV and 0.01 eV, respectively, identifying them as the most efficient HER catalysts. The H-Ni@B_40_ and H-Cu@B_40_ complexes further demonstrated a favorable hydrogen adsorption energy (ΔE_H*_) of -0.29 eV and − 0.25 eV, reinforcing their stability. Density of states (DOS) analysis revealed the formation of new energy states upon hydrogen adsorption, facilitating charge transfer between Ni@B_40_ and H, in agreement with frontier molecular orbital (FMO) analysis. These findings underscore the potential of TM@B_40_ complexes as highly efficient SACs for HER, offering a viable strategy for designing cost-effective and high-performance electrocatalysts for hydrogen production.

**Supplementary Information:**

The online version contains supplementary material available at 10.1038/s41598-026-46437-6.

## Introduction

The widespread reliance on fossil fuels has led to severe environmental consequences, including ozone depletion, escalating pollution levels, and accelerated global warming^1^. As global energy demand continues to rise, the development of sustainable energy alternatives has become imperative. In this context, hydrogen (H_2_) has emerged as a promising clean energy carrier capable of mitigating the environmental impact of fossil fuel consumption^2^, offering a viable pathway to reduce greenhouse gas emissions^3^. Among hydrogen production methods, electrocatalytic water splitting stands out as a key energy conversion process due to its potential for high efficiency and scalability^4^. However, the performance of this technology hinges on the availability of robust catalysts that can minimize energy barriers for water dissociation while maintaining long-term stability and high hydrogen evolution reaction (HER) activity^5^. Although some of the catalysts are designed and synthesized but these electrocatalytic hydrogen production faces significant challenges, including high desorption temperatures^6^, limited storage capacity, sluggish reaction kinetics^7^ and prohibitive material costs, all of which hinder large-scale implementation. Consequently, the design of advanced electrocatalysts remains critical to optimizing HER efficiency.

Several organic and inorganic nanomaterials have been designed and synthesized in the past few decades with promising catalytic properties. However, the organic nanomaterials have limitations of poor conductivity, biodegradability, difficulties in reproduction, poor thermal stability, and environmental and solvent sensitivity^8^. Recent efforts have explored various catalytic systems, such as cobalt diamine-dioxime complexes for acidic HER, monolayer-coated Ni complexes to minimize platinum usage, and ruthenium-based catalysts (e.g., RuP@NPC) exhibiting low overpotentials (10 mA cm⁻^2^) and 100% Faradaic efficiency with high stability^9^. Additionally, ultrathin WS₂ nanoflakes have demonstrated exceptional HER performance, achieving a Tafel slope of 48 mV/dec and an overpotential of ~ 100 mV under acidic conditions^10^. Mousavian and Esrafili designed a transition metal-doped C_24_N_24_ nanocage for methane oxidation and found that inorganic nanocages are more efficient for catalytic activity^11^. Moreover, gas sensing performance of Ni decorated inorganic aluminum/boron and nitride/phosphide nanocages showed promising results^12^. Subramani et al.. analyzed hydrogen storage capacities of organic C_24_ and inorganic aluminum and boron phosphide (Al/B_12_P_12_) nanocages and found enhanced potential for these cages doped with transition metals^13^.

Boron-based nanostructures have earned considerable attention in energy research due to their exceptional thermal stability^14,15^, lightweight nature^16,17^, and tunable electronic properties^18,19^. Recent studies have investigated a range of boron nanocages (e.g., B_6_, B_8_, B_36_, B_40_ and B_80_), with the D_*h*_-symmetric B_40_ nanocage exhibiting remarkable stability and electronic characteristics^20^. The integration of boron nanostructures with single-atom catalysts (SACs) presents a compelling strategy for enhancing HER performance, leveraging both the structural robustness of boron and the catalytic efficiency of SACs^21^. Wei et al.. designed transition metals doped boron nitride nanoclusters to investigate their HER activities over a wide range of pH. The V@B_5_N_3_ and Hf@B_2_N at neutral, alkaline and acidic environment were found to exhibit excellent HER response^22^. Furthermore, Ni- and Co-decorated B₁₂P₁₂ and Al₁₂P₁₂ complexes exhibited near-ideal Gibb’s free energies for HER, highlighting their potential as noble-metal-free alternatives^23^. These findings highlight the potential for designing highly efficient and cost-effective single-atom catalysts (SACs) based on transition metal-doped boron nanoclusters, with promising applications in the hydrogen evolution reaction (HER). Although numerous SACs have been developed and investigated, certain valuable inorganic nanomaterials, such as the B_40_ nanocage, remain largely unexplored for HER applications.

In this study, we employed density functional theory (DFT) to systematically evaluate the hydrogen evolution reaction (HER) catalytic performance of late first-row transition metal-decorated B_40_ complexes (TM@B_40_; TM = Zn, Fe, Co, Cu, Ni) as single-atom catalysts (SACs). Late first-row transition metals are frequently selected for HER investigations due to their favorable electronic configurations, moderate hydrogen intermediate binding energies, and natural abundance. An effective HER catalyst must exhibit an optimal hydrogen binding strength, neither too weak nor too strong to facilitate efficient adsorption and subsequent desorption of H* intermediates, as dictated by the Sabatier principle^24^. These late transition metals (TMs) often exhibit optimal d-band centers and catalytic behavior near this energetic balance, particularly when tailored through coordination tuning, alloying, environmental engineering, or doping. Furthermore, their ability to form stable active sites and diverse coordination complexes in heterogeneous catalysts enables the modulation of electronic structures to facilitate efficient H^+^ reduction. Notably, Ni- and Co-based nanomaterials have demonstrated competitive HER activity in both neutral and alkaline media, positioning them as promising non-precious alternatives to platinum-based catalysts^25^. These studies highlight the fundamental significance and practical relevance of TM-based SACs for sustainable hydrogen production.

## Research methodology

All calculations are performed by using Gaussian 09 software^26^, and results are visualized through visual builder’ GuassView 6.0 (All calculations are performed by using Gaussian 09 software, and results are visualized through visual builder’ GuassView 6.0 (https://gaussian.com/gaussview6/)^27^. Geometric structure of pure B40 is optimized by using the Beck three-parameter with Lee-Yang-Par exchange-correlation functional (B3LYP) with 6–31G(d) basis set. B3LYP is the most suitable functional of DFT that describes the geometrical parameters of fullerenes better than others^28^. The B3LYP functional of DFT with 6-31G (d) basis set has been used in literature for the determination of different properties, such as geometric, thermodynamic stability, electronic properties^29^. After optimization, frequency simulations are performed to confirm the true minimization of all the designed complexes, as indicated by obtaining only positive frequencies of all complexes without any imaginary frequency. The interaction (E_int_) of metals with B40 cage is calculated by using the Eq. 1^30^.

1 $$\:{E}_{int}=\:{E}_{{(B}_{40}\:nanocage+TM)}-({E}_{{B}_{40}\:nanocage}\:+\:{E}_{TM})$$

Here, $\:{E}_{{B}_{40}\:nanocage}$ represents B40 nanocage energy, E_TM_ is the energy of the individual Ni, Co, Cu, Fe and Zn metals and $\:{E}_{{(B}_{40}\:nanocage+TM)}\:$is the energy of B_40_ with the respective transition metal after optimization. Dispersion corrections play an important role in complexation reactions of two interacting species. In our study, we also analyzed the interaction of two different chemical species (B40 and TMs) to form complexes. So, we also calculated the interaction energy of all TM@B_40_ complexes at B3LYP-D3/6–31 + G (d) method, both in gas and aqueous phases. All the calculations in solvent (aqueous phase) are performed using conductor like polarizable continuum model (CPCM) as implemented in the Gaussian 09 software. CPCM is an implicit solvation model in which the solvent is represented as a polarizable dielectric continuum surrounding the solute cavity. The solute charge distribution polarizes the continuum, and the reaction field generated by the solvent is self-consistently included in the electronic structure calculation. For aqueous phase calculations, water is used as the solvent (dielectric constant = 78.3553). Regarding geometry optimization and energy evaluation: the geometries of the complexes are fully optimized in the solvent phase (water) using CPCM. The corresponding isolated fragments is also optimized in the same solvent enviroment to ensure methodological consistency. Interaction energies in solution are computed using the following approach:

2 $$\:{E}_{int}^{solv}=\:{E}_{complex}^{solv}-({E}_{fragement\:1}^{solv}+\:{E}_{fragment\:2}^{solv})$$

Here, $\:{E}_{int}^{solv}$, $\:{E}_{complex}^{solv}$, $\:{E}_{fragement\:1}^{solv}$and $\:{E}_{fragment\:2}^{solv}$ represent interaction energy of the complex in aqueous solvent, energy og of the complex, energy of fragment 1 (B_40_ nanocage) and energy of fragment 2 (transition metal in each corresponding transition meta doped B_40_ complexes). Where all energies are obtained using the same level of theory and CPCM solvation model. The same fragmentation scheme used for gas phase interaction energy calculations is consistently applied in the solvent phase calculations to allow direct comparison between gas and aqueous results. The B3LYP-D3 density functional has a dispersion correction function. Basis set’ 6–31 + G (d) having additional diffuse function so that we get more accurate and precise results for our designed complexes. Interaction energy of a system, especially transition metals containing complexes, is dependent on density functional and basis sets. To get accurate results, all the calculations were performed using additional PBE0-D3(BJ), M06, M06-L, ωB97X-D density functionals and Dunning basis sets (def2-SVP and def2-TZVP). PBE0-D3(BJ), M06, M06-L, ωB97X-D and B3LYP-D3 with def2-SVP are used for optimization of all complexes, and then single-point energies calculations are performed for all complexes using all density functionals with def2-TZVP in both gas and aqueous phases.

The amount of energy required to deform an isolated structure from its optimized geometry to the geometry it adopts in final complex after complexation is known as deformation energy. Deformation energy provides an indication of the structural strain induced by metal doping. Low deformation energy implies minimal distortion of the cage, thereby confirming the energetic favorability and stability of the doped configuration. In contrast, high deformation energy indicates significant distortion, energetic unfavourability and reduced stability of the doped configuration. In the present study, B_40_ nanocage is deformed by transition metal doping, whereas transition itself does not undergo geometric deformation. The effect of transition metals on B_40_ nanocage is therefore evaluated through the calculation of deformation energy^31^, as defined by Eq. 3.

3 $$\:{E}_{def}=\:{E}_{{deformedB}_{40}}-{E}_{{B}_{40}\:nanocage}$$

$\:{E}_{def}$ is deformation energy, obtained from the single point energy of B_40_ nanocage after removal of transition metal from TM@B_40_ complexes. Additionally, by subtracting the deformation energy from the interaction energy, the attraction energy for each complex is obtained. Frontier molecular orbital (FMO) analysis is performed to evaluate the electronic stability of designed complexes at B3LYP/6-31G(d) basis set. The energy difference (E_gap_) between the highest and lowest molecular orbitals, determined using Eq. 4^32^.

4 $${\mathrm{E}}_{{{\mathrm{gap}}}} = {\text{ E}}_{{{\mathrm{LUMO}}}} - {\text{ E}}_{{{\mathrm{HOMO}}}}$$

E_gap =_ difference between energies of HOMO-LUMO.

E_LUMO_ = Energy of lowest unoccupied molecular orbital.

E_HOMO =_ Energy of the highest occupied molecular orbital.

Natural Bond Orbital (NBO) charge and Mulliken charge analyses are performed at the same level of theory (B3LYP/6-31G(d)) to observe the charge transfer between the B_40_ cage and the considered transition metal (TM)^33^. Additionally, the density of states (DOS) analysis is calculated at quantum expresso^34^ using the same method (B3LYP/6-31G(d)) via Multiw*f*n software^35^, which provides the information about the energy states of occupied and unoccupied orbitals before and after the adsorption of transition metals on B_40_.

In theoretical studies, change in the Gibb’s free energy of hydrogen production (ΔG_H*_) serves as the primary activity descriptor for the hydrogen evolution reaction (HER). This HER activity descriptor (ΔG_H*_) provides insights into the catalyst’s performance. A highly positive ΔG_H*_ indicates unfavorable hydrogen adsorption, whereas a highly negative ΔG_H*_ suggests excessively strong hydrogen adsorption. In both cases, catalytic efficiency is hindered, as the former limits hydrogen binding while the latter impedes hydrogen desorption required for H₂ production. Thus, an ideal catalyst exhibits a ΔG_H*_ value as close to zero as possible^36^. The HER mechanism involves the following half-cell reaction pathway:

5 $${\mathrm{H}}^{ + } _{{({\mathrm{aq}})}} + {\text{ e}}^{ - } + {\text{ }}*{\text{ }}\to\frac{1}{2} {\mathrm{H}}_{{{\mathrm{2}}\left( {\mathrm{g}} \right)*}}$$

This is the Volmer step, which involves the adsorption of a proton and an electron transfer to form an adsorbed hydrogen atom on the catalyst surface. The H^+^, e^−^ and * are the notations for proton, electron and active site on the catalytic surface, respectively. The Heyrovsky step corresponds to electrochemical desorption, in which an adsorbed hydrogen atom reacts with a proton and an electron from the solution to form molecular hydrogen, as shown in Eq. 6:

6 $${\mathrm{H}}^{ + } _{{({\mathrm{aq}})}} + {\text{ H }}* + {\text{ e}}^{ - } ~~ \to ~{\mathrm{H}}_{{{\mathrm{2}}({\mathrm{g}})}} + {\text{ }}*$$

The Tafel step involves the chemical recombination of two adsorbed hydrogen atoms leading to the formation of molecular hydrogen, as described in Eq. 7:

7 $${\text{2H }}*~~~ \to {\mathrm{H}}_{{{\mathrm{2}}({\mathrm{g}})}} + *$$

According to the above equation, the Gibb’s free energy of the reactant is equal to the Gibb’s free energy of the product under standard conditions of temperature and pressure. The overall Gibb’s free energy of this reaction is considered to be zero. Typically, theoretical results for H₂ are used to estimate ΔG_H*_. Equation 8 shows the ΔG_H*_ as given below:

8 $$\Delta {\mathrm{G}}_{{{\mathrm{H}}*}} = {\text{ }}\Delta {\mathrm{E}}_{{{\mathrm{H}}*}} + {\text{ }}\Delta {\mathrm{E}}_{{{\mathrm{ZPE}}}} - {\text{ T}}\Delta {\mathrm{S}}_{{{\mathrm{H}}*}}$$

Whereas ∆E_H*_ is the adsorption of hydrogen energy change, and ∆E_ZPE_ is the change in zero-point energy of adsorbed hydrogen, T is the temperature 298.15 K, ∆S_H*_ is the entropy change after hydrogen adsorption. Equation 9 for the hydrogen adsorbed energy calculations^37^ is given below:

9 $$\Delta {\mathrm{E}}_{{{\mathrm{H}}*}} = E_{{(B_{{40}} ~nanocage + TM + H)}} - (E_{{(B_{{40}} ~nanocage + TM)}} + \frac{1}{2}{\mathrm{E}}_{{{\mathrm{H}}_{2} }} )$$

$\:{E}_{{(B}_{40}\:nanocage+TM+H)}$is the energy of hydrogen adsorption on transition metal doped on nanocage of B_40_, $\:{E}_{{(B}_{40}\:nanocage+TM)}$is the energy of _transition_ metals doped on B_40_ nanocage. $\:{\mathrm{E}}_{{\mathrm{H}}_{2}}$ is commonly known as the energy of hydrogen. Equation 10 is used for calculations of ∆E_ZPE_.

10 $$\Delta {\mathrm{E}}_{{{\mathrm{ZPE}}}} = E_{{ZPE(B_{{40}} ~nanocage + TM + H)~}} - (E_{{ZPE(B_{{40}} ~nanocage + TM)~}} + ~\frac{1}{2}{\mathrm{E}}_{{{\mathrm{ZPE}}\left( {{\mathrm{H}}_{2} } \right)}} )$$

$\:{E}_{ZPE{(B}_{40}\:nanocage+TM+H)\:}$is zero-point energy of the adsorbed hydrogen at the doped transition metal and $\:{E}_{ZPE{(B}_{40}\:nanocage+TM)\:}$ is commonly known as the energy of zero-point of doped B_40_ nanocage with transition metals and $\:{E}_{ZPE\left({H}_{2}\right)}$ is the zero-point energy of hydrogen that has been used. Equation 11 is used for calculation of ∆S_H*_^38^.

11 $$\Delta {\mathrm{S}}_{{{\mathrm{H}}*}} = S_{{(B_{{40}} ~nanocage + TM + H)~}} - (S_{{(B_{{40}} ~nanocage + TM)~}} + \frac{1}{2}S_{{H_{2} }} )$$

$\:{S}_{{(B}_{40}\:nanocage+TM+H)\:}$is the absolute entropy of the hydrogen adsorbed doped nanocage, $\:{S}_{{(B}_{40}\:nanocage+TM)\:}$ is the absolute entropy of transition metal doped B_40_ nanocage, and $\:{S}_{{H}_{2}}$ is known as hydrogen with absolute entropy. Gibb’s free energy (ΔG_H*_), zero-point energy and hydrogen adsorption energy (∆E_H*_) are calculated at both B3LYP/6-31G (d) and B3LYP-D3/6–31 + G (d) in the gaseous phase. Afterward, all calculations are also repeated in aqueous phase at B3LYP-D3/6–31 + G (d) to ensure the practical insights of HER processes.

The adsorption energies, including BSSE error corrections have also been estimated at B3LYP-D3/6-31G(d) level of theory in aqueous and gas phases. For this purpose, the counterpoise corrected (CP) calculations have been implemented for the correction of basis set superposition error (BSSE). The BSSE originated during complexation reactions when two chemical species interact with each other via weak forces. The idle basis functions of one specie interact with another one at short distances which results in this error. The equation of Boys and Bernardi^39^ is used for the calculation of this error as given below:

12 $${\mathrm{E}}_{{{\mathrm{cp}}}} = {\text{ E}}_{{{\mathrm{int}}}} /{\mathrm{E}}_{{{\mathrm{H}}*}} - {\text{ E}}_{{{\mathrm{BSSE}}}}$$

E_int_/E_H*_, E_cp._ and E_BSSE_ are symbols for interaction energy of TM@B_40_/adsorption energy of H-TM@B_40_, counterpoise corrected energy and energy of basis set superposition error, respectively.

## Results and discussion

**Fig. 1:**
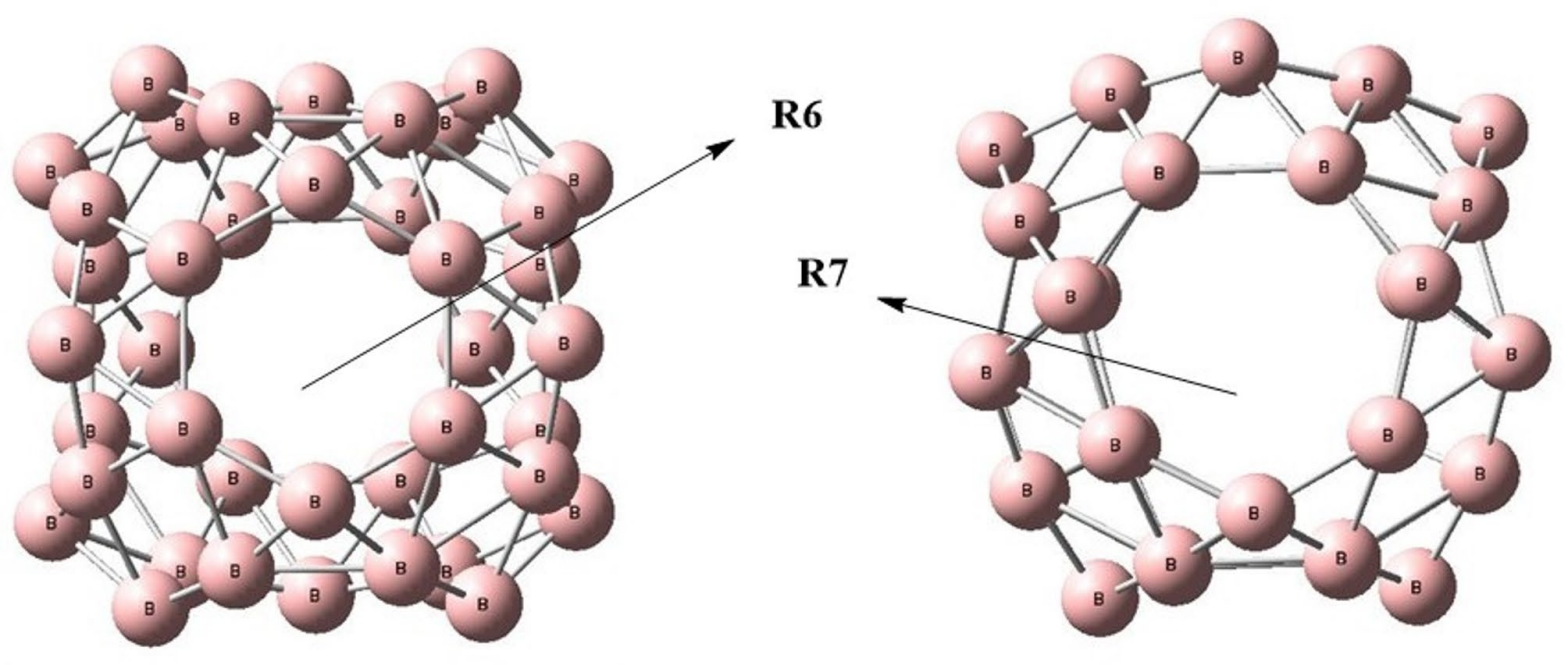
The optimized geometry of the B_40_ nanocage, highlighting the top of the hexagonal ring (R6) and the top of the heptagonal ring (R7) at B3LYP-D3 with 6–31 + G(d) basis set. All calculations are performed by using Gaussian 09 software, and results are visualized through visual builder’ GuassView 6.0 (https://gaussian.com/gaussview6/).

Initially, the isolated B_40_ cluster was geometrically optimized using density functional theory (DFT). The B_40_ structure comprises a combination of trigonal, tetragonal, pentagonal, hexagonal, and heptagonal rings, as previously reported^40^. Among the multiple potential binding sites on the B_40_ surface, the most favorable adsorption sites are located atop the six-membered (R6) and seven-membered (R7) rings, as illustrated in Fig. 1. These preferred binding sites are consistent with earlier computational and experimental studies^41^.

**Fig. 2:**
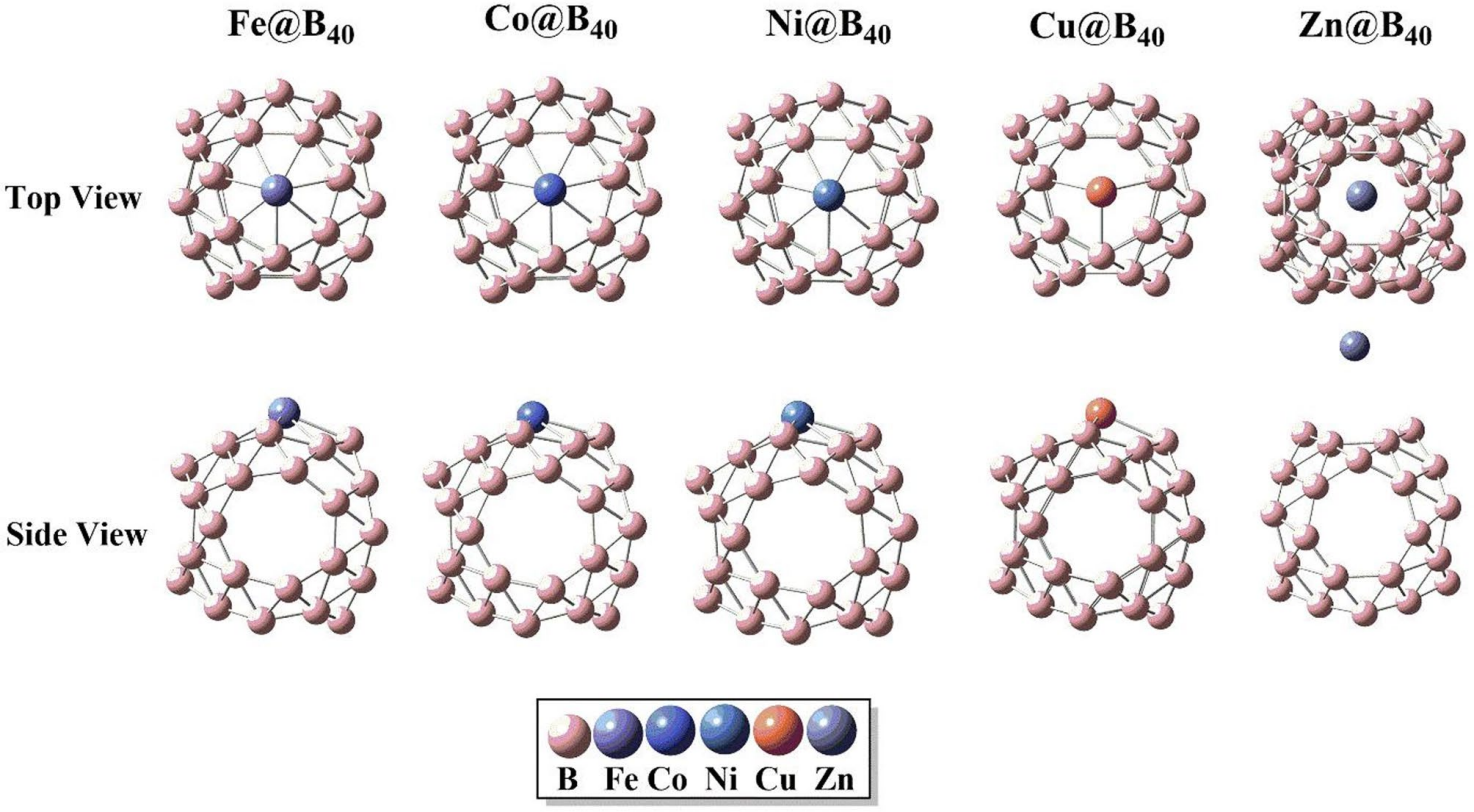
Optimize structures of transition metals (Zn, Fe, Cu, Co and Ni) adsorbed B_40_ complexes. All calculations are performed by using Gaussian 09 software, and results are visualized through visual builder’ GuassView 6.0 (https://gaussian.com/gaussview6/).

To investigate the site preference and electronic behavior of transition metal (TM) doping on the B₄₀ cluster, selected late first-row TMs—namely Zn, Fe, Co, Cu, and Ni—were adsorbed on two representative sites: the top of the hexagonal ring (R6) and the top of the heptagonal ring (R7). Following geometric optimization, it was observed that all TMs, except Zn, exhibit preferential binding at the R7 site. In contrast, Zn demonstrates greater structural stability at the R6 site, as illustrated in Fig. 2.

Given that these TMs possess partially filled valence *d*-orbitals, they are capable of existing in multiple spin configurations. To identify the ground-state spin multiplicity of each TM@B_40_ complex, spin-polarized calculations were performed, evaluating three plausible spin states for each system. The energetically most favorable spin state was then selected for subsequent analysis. The relative energies of these spin states are summarized in Table 1.

The most stable spin multiplicities for Fe@B_40_, Co@B_40_, Cu@B_40_, Ni@B_40_, and Zn@B_40_ were found to be triplet, doublet, triplet, doublet, and singlet, respectively. The second-lowest energy configurations correspond to the quintet, quartet, quintet, quartet, and triplet states, while the third most stable states were singlet, sextet, singlet, sextet, and quintet for the respective complexes. Notably, the relative energy differences between these spin states exceeded 10 kcal/mol in all cases, underscoring the importance of accurately accounting for spin polarization in modeling the electronic properties of TM-doped B_40_ systems.

**Table 1:** Relative energies (E_rel_ in kcal/mol) of different spin states of TM adsorbed B_40_ complexes.

Stable spin state	Fe@B_40_	Co@B_40_	Ni@B_40_	Cu@B_40_	Zn@B_40_
Most stable	Triplet	Doublet	Triplet	Doublet	Singlet
2nd stable	Quintet	Quartet	Quintet	Quartet	Triplet
3rd stable	Singlet	Sextet	Singlet	Sextet	Quintet
*E*_*rel*_ of most stable	0	0	0	0	0
*E*_*rel*_ of 2nd stable	12.72	22.03	39.53	43.05	27.56
*E*_*rel*_ of 3rd stable	20.27	56.94	152.74	194.56	73.79

The spin-state energetics of the TM@B_40_ complexes were systematically analyzed to identify the most thermodynamically stable configurations. For the Fe@B_40_ complex, the triplet spin state emerged as the ground state. The quintet and singlet configurations were found to be energetically less favorable, with relative energy differences (E_rel_) of 12.72 kcal/mol and 20.27 kcal/mol, respectively. In the case of Co@B_40_, the doublet state was identified as the most stable, whereas the quartet and sextet spin states were higher in energy by 22.03 kcal/mol and 56.94 kcal/mol, respectively. The Ni@B_40_ complex favored a triplet ground state, with the quintet and singlet configurations being less stable by 39.53 kcal/mol and 152.74 kcal/mol, respectively. For Cu@B_40_, the doublet state was energetically most favorable, while the quartet and sextet states were found to be 43.05 kcal/mol and 194.56 kcal/mol less stable, respectively. Finally, Zn@B_40_ exhibited a singlet ground state, with the triplet and quintet configurations being destabilized by 27.56 kcal/mol and 73.79 kcal/mol, respectively.

Boron is electron-deficient and has the potential to interact with electron-rich metal atoms. The strong interaction between the transition metals and the electron-deficient B_40_ framework is further evidenced by the short TM-B_40_ bond lengths, consistent with previous literature reports^42^. The computed interaction distances between the TM atom and the B₄₀ cage are 2.03 Å (Fe), 1.97 Å (Co), 1.99 Å (Cu), 2.43 Å (Zn), and 2.02 Å (Ni), as detailed in Table 2.

The thermal stability of the TM@B_40_ complexes was evaluated through interaction energy (E_int_) calculations. All systems exhibited negative E_int_ values, indicative of spontaneous complex formation and thermodynamic stability. At the B3LYP/6-31G(d) level, the E_int_ magnitudes follow the trend: Fe@B_40_ (–7.25 eV) > Co@B_40_ (–6.57 eV) > Cu@B_40_ (–4.57 eV) > Ni@B_40_ (–2.20 eV) > Zn@B_40_ (–1.09 eV). Notably, Fe@B_40_ demonstrates the most exergonic binding energy, underscoring its superior thermodynamic stability. In contrast, at the B3LYP-D3/6–31 + G(d) level, the trend shifts to: Co@B_40_ (–3.51 eV) > Fe@B_40_ (–3.48 eV) > Ni@B_40_ (–2.64 eV) > Cu@B_40_ (–1.88 eV) > Zn@B_40_ (–0.36 eV). The higher E_int_ values obtained with B3LYP/6-31G(d) compared to B3LYP-D3/6–31 + G(d) likely arise from the absence of dispersion corrections in the former, alongside the improved description of occupied molecular orbitals by diffuse functions in the latter basis set.

To further elucidate stabilization mechanisms, E_int_ was decomposed into stabilizing attraction and destabilizing deformation energies. The Zn@B_40_ complex exhibits the lowest deformation energy of 0.09 eV, followed by Co@B_40_ (0.33 eV) and then lastly Cu@B_40_ (0.38 eV), rationalizing its high E_int_ (–3.51 eV) at the B3LYP-D3/6–31 + G(d) level. For Co@B_40_, Cu@B_40_ and Zn@B_40_, deformation energies remain below 1 eV, while attraction energies exceed 2 eV, attributed to the TM atoms (Co, Cu, Zn) being stabilized within the seven-membered aromatic ring cavities of the B_40_ nanocage. Conversely, Fe@B_40_ shows the strongest attraction energy (E_att_ = − 5.83 eV), consistent with its large E_int_ (–3.48 eV), followed by Ni@B_40_ having E_att_ of -4.86 eV and large E_int_ (-2.64 eV). The attraction energy represents pure electronic stabilization arising from the doping of transition metal on B_40_ cage excluding deformation energy and it is used asses the intrinsic strength of TMs doped cage binding. The larger attraction energy value indicates the stronger TMs and cage interaction as seen in case of Fe@B_40_ and Ni@B_40_ complexes, confirming their stability.

In aqueous phase (B3LYP-D3/6–31 + G(d)), E_int_ values increase to − 3.72 (Fe@B_40_), − 3.63 (Co@B_40_), − 2.83 (Ni@B_40_), − 1.94 (Cu@B_40_), and − 1.16 eV (Zn@B_40_), indicating enhanced thermodynamic stability due to solvation effects. These trends align with prior reports on transition metal-doped boron clusters, supporting their potential applicability^43^. Counterpoise-corrected energies (E_cp._) in the gas phase range from − 0.31 to − 3.65 eV (B3LYP-D3/6–31 + G(d)), mirroring E_int_ trends, with negligible solvent-phase deviations except for Zn@B_40_.

For greater accuracy, all the complexes are optimized at PBE0-D3(BJ), M06, M06-L, ωB97X-D and B3LYP-D3 with def2-SVP (optimization), and single-point energies are calculated with def2-TZVP in both the gas phase and aqueous phase. The E_int_ values are calculated using all these methods and the corresponding E_int_ results are given in Table S1. All complexes show thermal stability at these selected methods (PBE0-D3(BJ), M06, M06-L, and/or ωB97X-D and B3LYP-D3 with def2-TZVP) which is confirmed by the negative E_int_ values. Among all methods, the higher E_int_ values are obtained using M06-L/def2-TZVP method for all complexes. The E_int_ values of Fe@B_40_, Co@B_40_, Ni@B_40_ Cu@B_40_ and Zn@B_40_ are − 8.13, − 5.54, − 5.51, − 3.22 and − 0.47 eV, respectively. Ni@B_40_ and Cu@B_40_ complexes have E_int_ of -5.35 and − 3.01 eV, respectively, at the M06-L/def2-TZVP method in the aqueous phase.

### Electronic properties of TM@B_40_ complexes

The electronic properties of the TM@B_40_ complexes (TM = Zn, Fe, Cu, Co, and Ni) were systematically investigated using Frontier Molecular Orbital (FMO) analysis to understand the influence of transition metal doping on the electronic structure of the B₄₀ nanocage. The incorporation of transition metal atoms induces notable modifications in the electronic characteristics of the B_40_ framework. Key electronic descriptors—including the energies of the highest occupied molecular orbital (HOMO), the lowest unoccupied molecular orbital (LUMO), and the HOMO–LUMO energy gap (E_gap_) were computed, and the corresponding values are listed in Table 2.

The calculated HOMO energies for Fe@B_40_, Co@B_40_, Ni@B_40_, Cu@B_40_, and Zn@B_40_ are − 7.58, − 8.13, − 7.54, − 6.85, and − 7.60 eV, respectively. The corresponding LUMO energies are − 7.40, − 6.52, − 6.78, − 6.55, and − 6.73 eV, respectively. The HOMO–LUMO energy gap is a critical parameter governing the electronic behavior of molecular systems. A reduced E_gap_ typically corresponds to increased electrical conductivity and reduced kinetic stability, while a larger gap suggests greater stability and reduced conductivity^44^.

The pristine B_40_ nanocage exhibits an E_gap_ of 2.93 eV, indicating its semiconducting nature. Upon doping with transition metals, a significant narrowing of the energy gap is observed. The trend in decreasing E_gap_ for the TM@B_40_ complexes follows the order: Co@B_40_ (1.60 eV) > Zn@B_40_ (0.87 eV) > Ni@B_40_ (0.75 eV) > Cu@B_40_ (0.28 eV) > Fe@B_40_ (0.18 eV). This reduction is primarily attributed to an upward shift in HOMO energy levels and a concurrent downward shift in LUMO energies upon TM interaction, a behavior previously documented in related systems^45^. The vertical electron affinity (2.90 eV) of the pure B_40_ nanocage at B3LYP is almost comparable to the experimental and theoretically reported vertical electron affinity (~ 2.50 eV) of B_40_^46^.

**Fig. 3:**
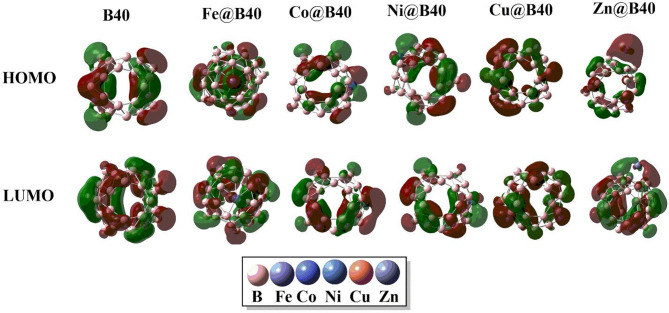
Graphics dispersion of isodensities of HOMOs and LUMOs of B_40,_ Fe@B_40_, Cu@B_40,_ Co@B_40,_ Ni@B_40,_ and Zn@B_40_ complexes. All calculations are performed by using Gaussian 09 software, and results are visualized through visual builder’ GuassView 6.0 (https://gaussian.com/gaussview6/).

Figure 3 visualizes the spatial distribution of the HOMO and LUMO isodensities for the pristine B_40_ nanocage and the TM-doped B_40_ complexes. In the undoped B_40_, HOMO electron density is predominantly localized along the B–B bonds and boron atoms, while the LUMO isodensity is distributed over individual boron sites. In contrast, for the TM@B_40_ systems, the HOMO isodensity shifts significantly towards the transition metal atoms, indicating their dominant contribution to the occupied frontier orbitals. The LUMO, however, remains primarily distributed over the boron atoms distant from the TM binding site, although some localization on boron atoms adjacent to the metal site is also observed, suggesting electronic coupling between the TM and the B_40_ framework.

To further quantify the electronic interactions, Natural Bond Orbital (NBO) and Mulliken charge analyses are conducted to estimate the charge transfer between the dopant metals and the B₄₀ nanocage. For Fe and Zn metals, the Mulliken and NBO charge analyses yield comparable results; however, different charge-transfer behavior is observed for the remaining metals (Co, Ni and Cu). The calculated NBO charges on the transition metal atoms in Fe@B_40_, Co@B_40_, Ni@B_40_, Cu@B_40_, and Zn@B_40_ are + 0.76 |e|, + 0.57 |e|, + 0.68 |e|, + 0.80 |e|, and + 0.50 |e|, respectively, as listed in Table 2. All NBO charge values are positive, indicating electron donation from the transition metal to the B₄₀ nanocage. In contrast, the corresponding Mulliken charges for Fe@B_40_, Co@B_40_, Ni@B_40_, Cu@B_40_, and Zn@B_40_ are + 0.53 |e|, − 0.03 |e|, − 0.09 |e|, − 0.07 |e|, and + 0.21 |e|, respectively. Positive Mulliken charges for Fe and Zn confirm electron donation to the B₄₀ nanocage, whereas negative values for Co, Cu and Ni indicate charge transfer from the B_40_ nanocage to the dopant metal. These results are consistent with previously reported studies on TM-boron cluster interactions, which demonstrate that charge redistribution depends on the metal’s electronegativity and coordination environment.

**Table 2:** The energy of LUMOs (E_L_), the energy of HOMOs (E_H_), the HOMO-LUMO energy gap (E_gap_), NBO charges on transition metals (Q_TM_), Mulliken charges (Q_TM−Mu_), bond length of TM-B_40_ (b_TM−B_) in and interaction energy (E_int_ in eV) of pure B_40_ and TM@B_40_ (TM = Fe, Co, Ni, Cu, Zn) complexes.

Complexes	E_L_ (eV)	E_H_ (eV)	E_gap_ (eV)	Q_TM_ |e|	Q_TM−Mu_ |e|	b_TM−B_ (Å)	E_int_ (eV)
Pure-B_40_	-2.90	-5.83	2.93	-	-	-	-
Fe@B_40_	-7.40	-7.58	0.18	0.53	0.76	2.03	-7.25
Co@B_40_	-6.52	-8.13	1.60	-0.03	0.57	1.97	-6.57
Ni@B_40_	-6.78	-7.54	0.75	-0.09	0.68	2.02	-2.20
Cu@B_40_	-6.55	-6.85	0.28	-0.07	0.80	1.99	-4.57
Zn@B_40_	-6.73	-7.60	0.87	0.21	0.50	2.43	-1.09

### Optimized geometries of H adsorption on TM@B_40_ complexes (TM = Fe, Co, Ni, Cu, Zn)

**Fig. 4:**
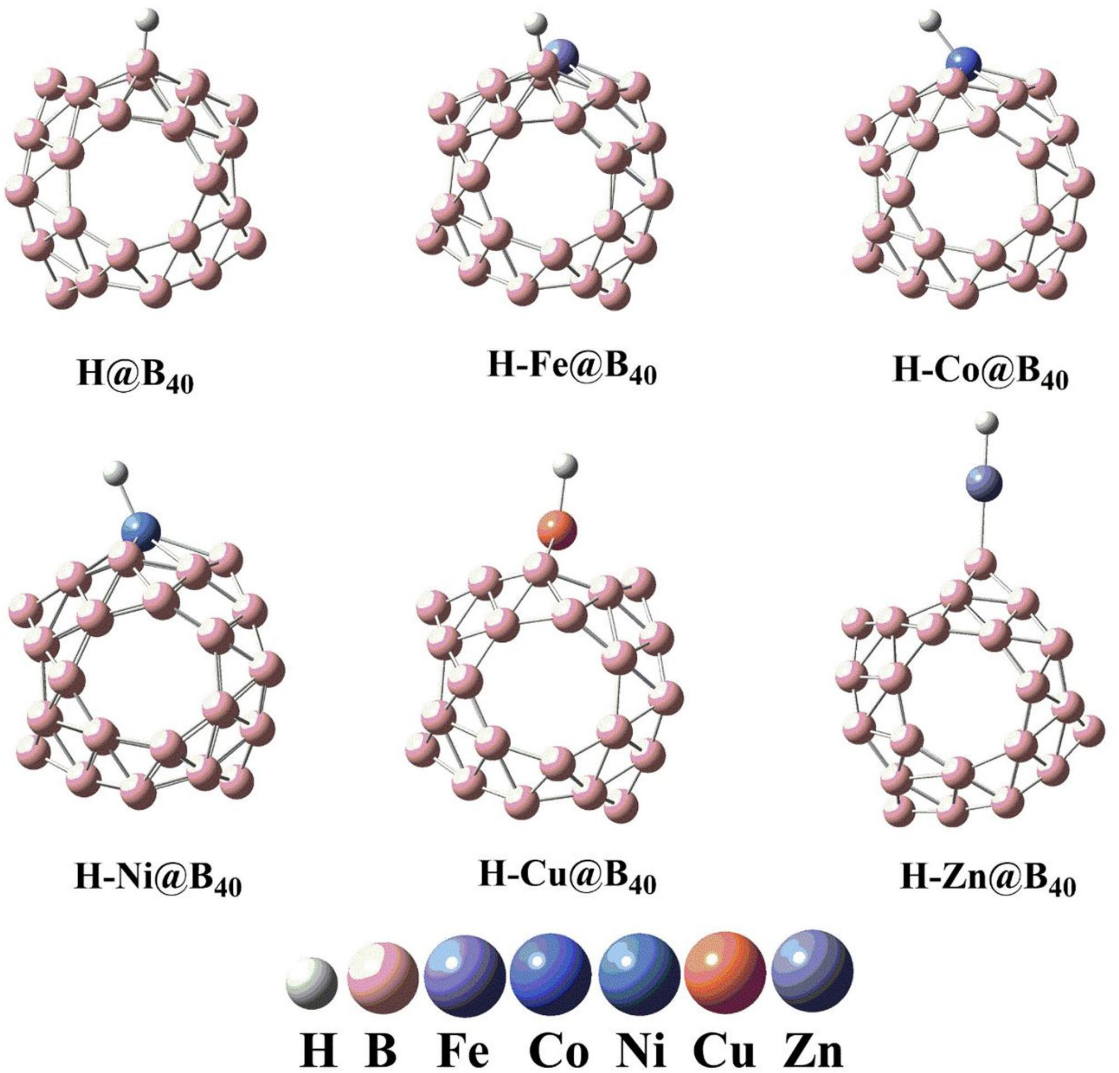
The optimized geometries of hydrogen adsorbed TM@B_40_ (TM = Fe, Co, Cu, Ni and Zn) complexes. All calculations are performed by using Gaussian 09 software, and results are visualized through visual builder’ GuassView 6.0 (https://gaussian.com/gaussview6/).

To evaluate the hydrogen evolution reaction (HER) catalytic potential of the studied systems, we examined hydrogen atom adsorption on pristine B_40_ and transition metal-decorated TM@B_40_ complexes (optimized geometries presented in Fig. 4).

Transition metals exhibit an inherently electropositive character, enabling them to induce significant polarity in adjacent species. Our geometric optimizations revealed a hydrogen-boron bond distance of 1.20 Å in the H@B_40_ system. For the transition metal-containing complexes, the hydrogen-metal bond distances were measured as 2.78 Å (Fe@B_40_), 1.46 Å (Co@B_40_), 1.54 Å (Cu@B_40_), 1.52 Å (Ni@B_40_), and 1.54 Å (Zn@B_40_), as detailed in Table 3. The comparatively shorter bond lengths observed in most TM@B_40_ systems confirm strong hydrogen interaction with the transition metal centers. Notably, while hydrogen preferentially binds to the transition metal sites in Co@B_40_, Ni@B_40_, Cu@B_40_, and Zn@B_40_ complexes, the H-Fe@B_40_ system exhibits distinct behavior, with hydrogen stabilization occurring at a boron atom of the B_40_ cage rather than the iron center.

The hydrogen interaction energies (E_H*_) presented in Table 3 reveal distinct thermodynamic behaviors among the studied systems, with values of -0.12 eV (Fe@B_40_), 0.54 eV (Co@B_40_), -0.26 eV (Ni@B_40_), 0.05 eV (Cu@B_40_), and 0.14 eV (Zn@B_40_) at B3LYP/6-31G (d) method. The negative interaction energy values observed for Fe@B_40_ and Ni@B_40_ systems indicate thermodynamically favorable, exothermic hydrogen adsorption processes. In contrast, the positive values for Co@B_40_, Cu@B_40_, and Zn@B_40_ suggest endothermic interactions, highlighting the superior stability and hydrogen evolution potential of Fe@B_40_ and Ni@B_40_ complexes. These findings align with established principles of catalytic surface interactions, where lower energy values correlate with stronger metal-hydrogen bonding and more stable SAC configurations^47^. Notably, pristine B₄₀ exhibits an E_H*_ of -0.03 eV, demonstrating that transition metal doping provides more favorable active sites for hydrogen adsorption. The hydrogen adsorption energies (E_H*_) for B_40_ and TM@B_40_ complexes (TM = Fe, Co, Ni, Cu, Zn) were computed at the B3LYP-D3/6–31 + G(d) level. In the gas phase, E_H*_ values are − 0.07 eV (B_40_), 0.29 eV (Fe@B_40_), 0.45 eV (Co@B_40_), − 0.39 eV (Ni@B_40_), − 0.07 eV (Cu@B_40_), and 0.20 eV (Zn@B_40_). The inclusion of dispersion corrections in B3LYP-D3 leads to a systematic increase in E_H*_ compared to uncorrected B3LYP, attributable to the more accurate treatment of van der Waals interactions.

In aqueous phase, E_H_* values shift to − 0.08 eV (B_40_), 0.61 eV (Fe@B_40_), 0.54 eV (Co@B_40_), − 0.29 eV (Ni@B_40_), − 0.25 eV (Cu@B_40_), and 0.31 eV (Zn@B_40_) at B3LYP-D3/6–31 + G(d) method. Solvation generally enhances hydrogen adsorption, except for Cu@B_40_, which exhibits a pronounced decrease in E_H_* (− 0.25 eV), suggesting unique solvent-induced electronic reorganization at the Cu active site. Counterpoise-corrected energies (E_cp._) for H-TM@B_40_ complexes in the gas phase range from − 0.31 to − 3.65 eV, mirroring trends observed for E_H*_. These E_cp._ values remain largely consistent in the aqueous phase, with deviations only for Cu@B_40_ and Zn@B_40_, further corroborating the solvent-dependent behavior of these systems.

These complexes show a variation trend of E_H_*at PBE0-D3(BJ), M06, M06-L, ωB97X-D and B3LYP-D3 with def2-TZVP methods in the gas phase. H-Fe@B_40_, H-Ni@B_40_ and H-Zn@B_40_ complexes show negative E_H_* values and the other complexes show positive E_H_* values at most of the method (see Table S1). The E_H_* values of H-Fe@B_40_, H-Co@B_40_, H-Ni@B_40_, H-Cu@B_40_ and H-Zn@B_40_ are 0.43, 0.36, − 0.30, 0.09 eV and − 0.09 eV at M06-L/def2-TZVP method. The same complexes show a decrease in the E_H_* values in the aqueous phase, where H-Ni@B_40_ and H-Cu@B_40_ complexes have E_H_* values of -0.29 and 0.10 eV, respectively.

Structural stability was further investigated through zero-point energy (ZPE) calculations following hydrogen adsorption. The obtained ZPE values of 0.06 eV (Fe@B_40_), 0.06 eV (Co@B_40_), 0.07 eV (Ni@B_40_), 0.06 eV (Cu@B_40_), and − 0.03 eV (Zn@B_40_) indicate that Zn@B_40_ exhibits particularly favorable vibrational characteristics for HER applications. Thermodynamic analysis at standard conditions (298.15 K, 1 atm) yielded entropy values of 158.55 eV (Fe@B_40_), 156.06 eV (Co@B_40_), 159.20 eV (Ni@B_40_), 159.84 eV (Cu@B_40_), and 164.08 eV (Zn@B_40_), with Co@B_40_ displaying the lowest entropy among the series, reflecting its more ordered configuration.

### Electronic properties of adsorb H-TM@B_40_ complexes

Frontier Molecular Orbital (FMO) analysis was further extended to investigate the electronic properties of the hydrogenated B_40_ nanocage (H@B_40_) and hydrogen-adsorbed TM-doped B_40_ complexes (H–TM@B_40_; TM = Fe, Co, Ni, Cu, Zn). The adsorption of a hydrogen atom on TM@B_40_ systems results in noticeable variations in the HOMO, LUMO, and HOMO–LUMO energy gap (E_gap_) values. The calculated HOMO energies for H–Fe@B_40_, H–Co@B_40_, H–Ni@B_40_, H–Cu@B_40_, and H–Zn@B_40_ are − 7.83, − 8.18, − 7.60, − 6.73, and − 8.76 eV, respectively. The corresponding LUMO energies are − 7.55, − 7.00, − 7.22, − 6.58, and − 7.06 eV, respectively, as summarized in Table 3.

The resulting HOMO–LUMO energy gaps follow the trend: H–Cu@B_40_ (0.15 eV) < H–Fe@B_40_ (0.28 eV) < H–Ni@B_40_ (0.38 eV) < H–Co@B_40_ (1.18 eV) < H–Zn@B_40_ (1.70 eV). Compared to the unhydrogenated TM@B_40_ complexes, the E_gap_ decreases upon hydrogen adsorption in the cases of H–Cu@B_40_, H–Co@B_40_, and H–Ni@B_40_, suggesting enhanced electrical conductivity and reduced kinetic stability. In contrast, an increase in E_gap_ is observed for H–Fe@B_40_ and H–Zn@B_40_, implying a decrease in electrical conductivity and greater kinetic stability. These variations in E_gap_ indicate that hydrogen adsorption significantly modifies the electronic structure and conductive properties of the TM@B_40_ complexes.

**Fig. 5:**
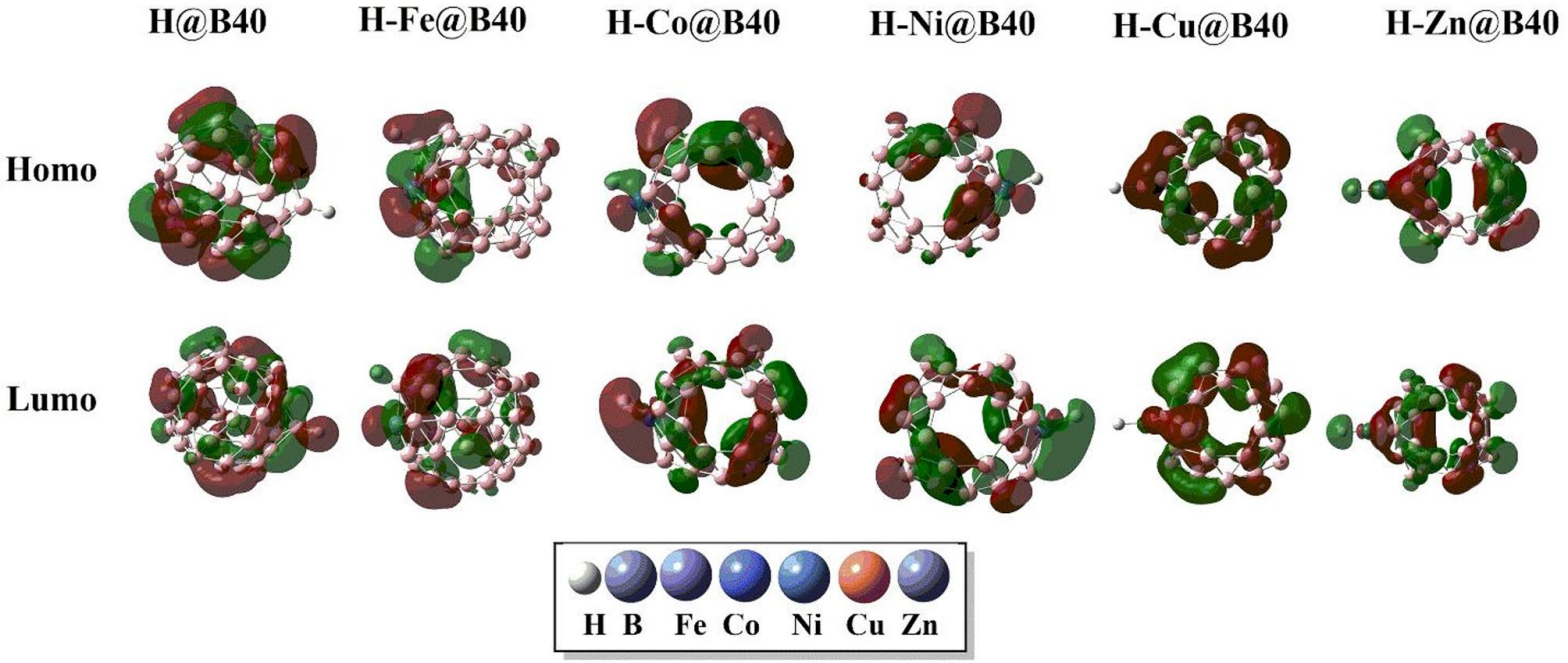
Graphics isodensities of HOMO and LUMO orbitals of H-TMs@B_40_ complexes. All calculations are performed by using Gaussian 09 software, and results are visualized through visual builder’ GuassView 6.0 (https://gaussian.com/gaussview6/).

The spatial distribution of HOMO and LUMO isodensities for H@B_40_ and H–TM@B_40_ systems is illustrated in Fig. 5. In the H@B_40_ complex, both HOMO and LUMO densities are uniformly distributed across the B_40_ cage, with a minor portion of LUMO density appearing on the hydrogen atom. Upon transition metal doping, the HOMO densities become localized primarily on the transition metal atom and the hydrogen atom, while some HOMO contributions are also observed on the boron atoms directly interacting with the dopants. The LUMO densities, however, are delocalized over boron atoms situated away from the metal and hydrogen interaction sites, reflecting spatial separation between the occupied and unoccupied frontier orbitals.

To further elucidate the electronic interactions between the hydrogen atom and the TM@B_40_ complexes, Natural Bond Orbital (NBO) and Mulliken charge analyses are performed. The Mulliken charges on the transition metal atoms in H–Fe@B_40_, H–Co@B_40_, H–Ni@B_40_, H–Cu@B_40_, and H–Zn@B_40_ are calculated to be + 0.42 |e|, − 0.19 |e|, − 0.31 |e|, − 0.24 |e|, and 0.25 |e|, respectively (Table 3). Positive Mulliken charges indicate net electron transfer from the transition metal to the hydrogen atom, as observed in the Fe and Zn doped complexes. In contrast, negative Mulliken charges for Co, Ni and Cu suggest electron donation from hydrogen to the corresponding TM@B_40_ frameworks. Alternatively, a positive NBO charge on each of the transition metal indicates a net electron transfer from the transition metal to the hydrogen atom.

The Mulliken and NBO charges on the hydrogen atom after adsorption further support this trend. The Mulliken charge values on hydrogen in H@B_40_, H–Fe@B_40_, H–Co@B_40_, H–Ni@B_40_, H–Cu@B_40_, and H–Zn@B_40_ are + 0.02 |e|, + 0.04 |e|, + 0.03 |e|, − 0.02 |e|, − 0.04 |e|, and − 0.04 |e|, respectively. Similarly, the corresponding NBO charge values on same hydrogen are H@B_40_, H–Fe@B_40_, H–Co@B_40_, H–Ni@B_40_, H–Cu@B_40_, and H–Zn@B_40_ are + 0.10 |e|, + 0.10 |e|, + 0.06 |e|, − 0.07 |e|, − 0.20 |e|, and − 0.32 |e|, respectively. These results reveal that in complexes such as H@B_40_, H–Fe@B_40_, and H–Co@B_40_, the hydrogen atom donates electronic density to the surrounding TM@B_40_ framework. Conversely, in H–Ni@B_40_, H–Cu@B_40_, and H–Zn@B_40_, the hydrogen atom gains electronic density, consistent with the direction of charge transfer inferred from the NBO analysis. It is worth noting that NBO charge analysis is generally more reliable than Mulliken charge analysis. Mulliken charges are highly sensitive to the choice of basis set and may exhibit sign reversal with changes in basis functions. In contrast, NBO charges are less basis set dependent, as they are derived from orthogonal localized orbitals, and therefore provide more robust and physically meaningful trend. Overall, this detailed examination of charge redistribution offers valuable insight into the electronic behavior of hydrogen-adsorbed TM@B_40_ complexes and their potential catalytic applications.

**Table 3:** Mulliken charges on hydrogen (Q_H−Mu_), NBO charge on metal (Q_TM_), NBO charge on H, Mulliken charges on transition metals (Q_Mu_), adsorption energy of H interacting with TM@B_40_ (E_H_), Gibb’s free energy of H adsorption (∆G_H_), HOMO-LUMO energy gap (E_gap_), the energies of LUMOs (E_LUMO_), the energies of HOMOs (E_HOMO_), and bond length between H and TM of TM@B_40_ (b_H−TM_) of the hydrogen adsorbed TM@B_40_ complexes (TM = Fe, Co, Ni, Cu, Zn).

Complexes	Q_TM−Mu_ |e|	Q_H−Mu_ |e|	Q_H_ |e|	Q_TM_ |e|	E_H_ (eV)	∆G_H_ (eV)	E_gap_ (eV)	E_LUMO_ (eV)	E_HOMO_ (eV)	b_H−TM_ (Å)
H-B_40_	-	0.02	0.10	-	-0.03	0.18	0.62	-8.12	-8.74	1.20
H-Fe@B_40_	0.42	0.04	0.10	0.73	-0.12	0.13	0.28	-7.55	-7.83	2.78
H-Co@B_40_	-0.19	0.03	0.06	0.23	0.54	0.80	1.18	-7.00	-8.18	1.46
H-Ni@B_40_	-0.31	-0.02	-0.07	0.33	-0.26	⁓0.003	0.38	-7.22	-7.60	1.52
H-Cu@B_40_	-0.24	-0.04	-0.20	0.48	0.05	0.30	0.15	-6.58	-6.73	1.54
H-Zn@B_40_	0.25	-0.04	-0.32	1.03	0.14	0.29	1.70	-7.06	-8.76	1.54

**Fig. 6:**
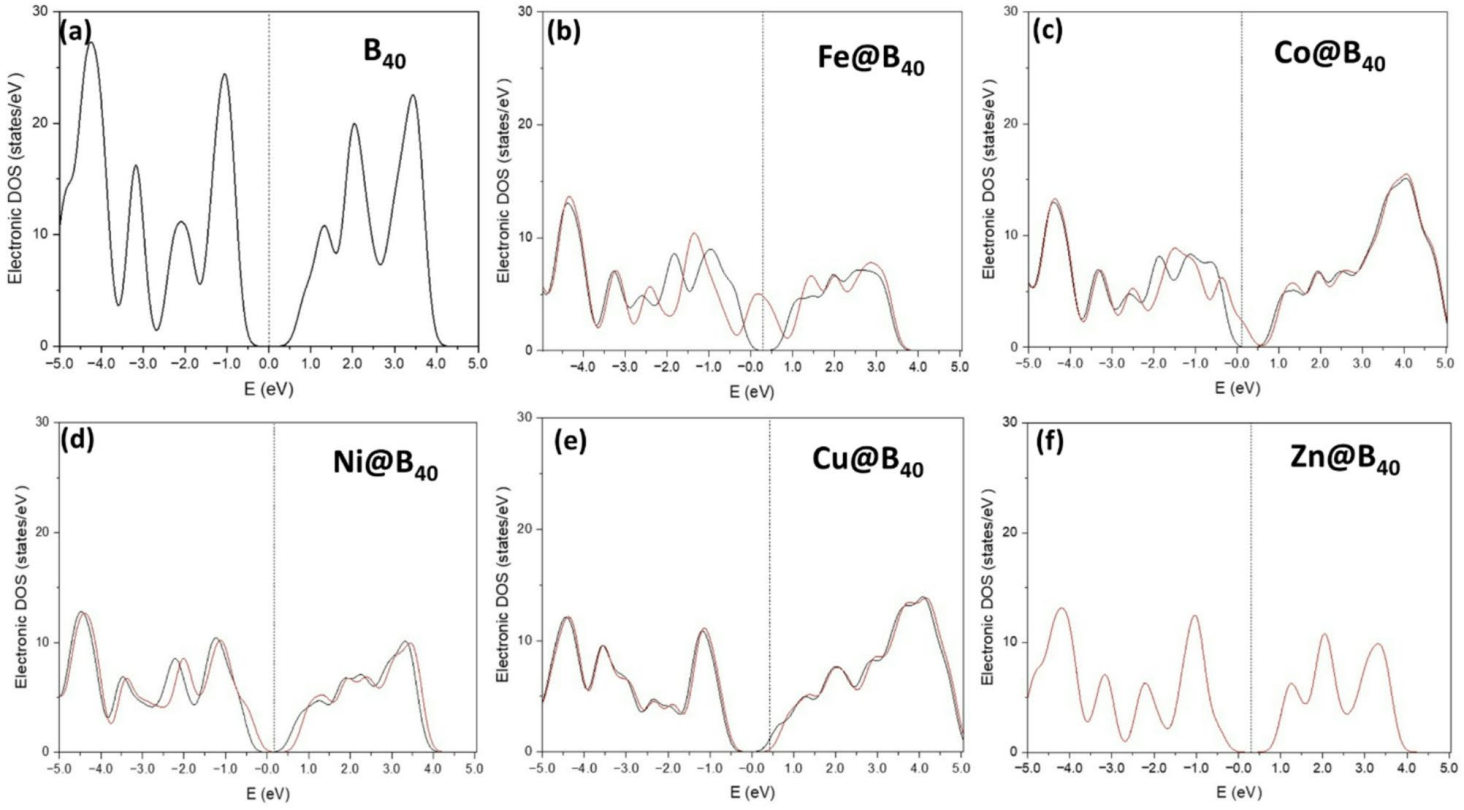
**a**-**f** The density of states (DOS) spectra of TM@B_40_ complexes.

The projected density of states (PDOS) analysis reveals significant electronic structure modifications upon transition metal doping in B_40_ systems, as shown in Fig. 6. Pristine B_40_ exhibits a distinct bandgap at the Fermi level, confirming its semiconducting nature, in agreement with previous theoretical studies^48^. This electronic configuration undergoes substantial transformation when doped with transition metals (Fe, Co, Ni, Cu, Zn), primarily through hybridization between the metal d-orbitals and boron p-orbitals.

The early transition metal-doped systems (Fe, Co, Ni) demonstrate marked changes in their electronic structure near the Fermi level. Fe@B_40_ shows Fermi level penetration into the valence band, indicative of semi-metallic character. Both Co@B_40_ and Ni@B_40_ exhibit pronounced density of states at the Fermi level, confirming metallic behavior resulting from strong d-p hybridization and partial occupation of d-states^49^. These electronic modifications are characterized by broadened electronic bands and the emergence of delocalized states, which enhance electronic conductivity, which is a crucial factor for catalytic applications. In contrast, late transition metal dopants (Cu, Zn) produce qualitatively different effects. Cu@B_40_ displays a broad DOS feature with the Fermi level positioned at its shoulder, reflecting the filled nature of Cu 3d orbitals. In Zn@B₄₀, Zn possesses a closed-shell 3d¹⁰ configuration, and its d orbitals are weakly hybridized with the B-2p states near the Fermi level; consequently, the Zn-related states either lie far from the frontier region or overlap with B states, resulting in a single dominant spectrum. This does not imply that Zn or B orbitals do not contribute, but rather that their states are energetically merged. This systematic variation in electronic properties correlates directly with the d-electron configuration of the dopant metals.

**Fig. 7:**
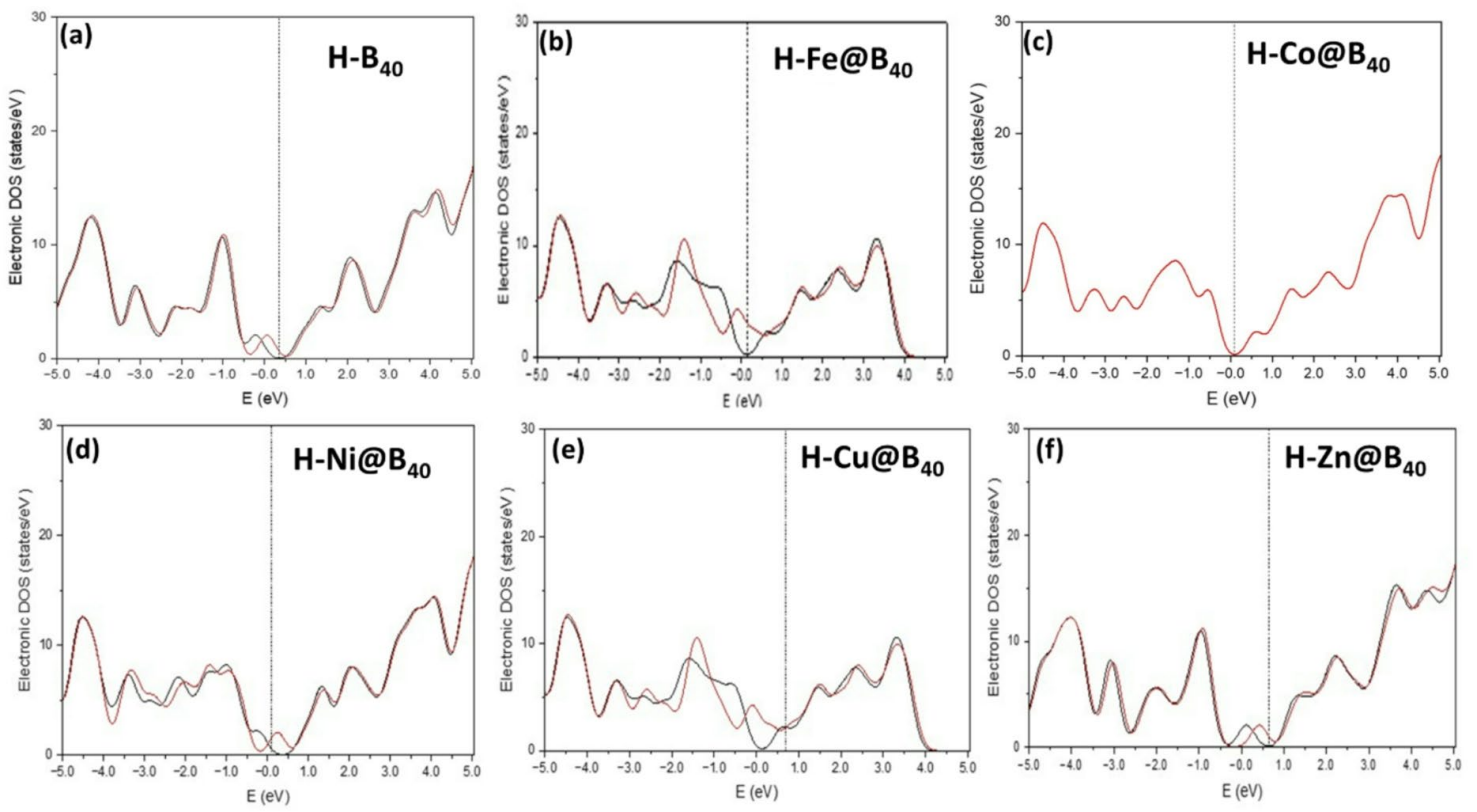
**a**-**f** The density of states (DOS) spectra of H doped TM@B_40_ complexes.

The hydrogen-adsorbed systems (H-TM@B_40_) show further electronic structure modifications with important implications for hydrogen evolution reaction (HER) activity (Fig. 7). While H-B_40_ maintains its semiconducting gap, limiting its catalytic potential, several doped systems exhibit metallic or semi-metallic characteristics favorable for electrocatalysis. H-Fe@B_40_ demonstrates spin-polarized metallic behavior, while H-Cu@B_40_ shows a particularly high density of states at the Fermi level, suggesting excellent charge transport properties. H-Co@B_40_ and H-Ni@B_40_ exhibit intermediate behavior with a moderate density of states at the Fermi level. Notably, H-Zn@B_40_ reverts to semiconducting behavior. Upon H adsorption, the H-1s orbitals interacts with Zn and B states, perturbing the electronic structure and lifting degeneracies, which leads to a clearer energetic separation and the appearance of two spectra. However, in H–Co@B₄₀, strong Co–H–B hybridization redistributes and delocalizes the Co-d states, causing them to overlap significantly with the B40 states and collapse into a single apparent spectrum.

These results establish clear structure-property relationships between transition metal doping and electronic characteristics in B_40_ systems. The induced metallicity in Fe-, Co-, and Ni-doped systems, contrasted with the maintained semiconducting behavior in Zn-doped systems, provides fundamental insights for designing boron-based electrocatalysts. The direct correlation between electronic structure modifications and predicted catalytic activity underscores the importance of electronic structure engineering in developing efficient HER catalysts.

### Identification and characterization of the binding orbitals

The interaction between transition metals (TMs) and the B_40_ nanocage is mediated through hybridization of TM d-orbitals with the available orbitals of the boron framework. These binding characteristics fundamentally govern the catalytic properties of the resulting TM@B_40_ complexes. To elucidate the nature of these interactions, we systematically investigated the molecular orbitals responsible for TM-B_40_ binding using non-periodic DFT calculations, employing both Mulliken population analysis and natural atomic orbital (NAO) decomposition.

**Fig. 8:**
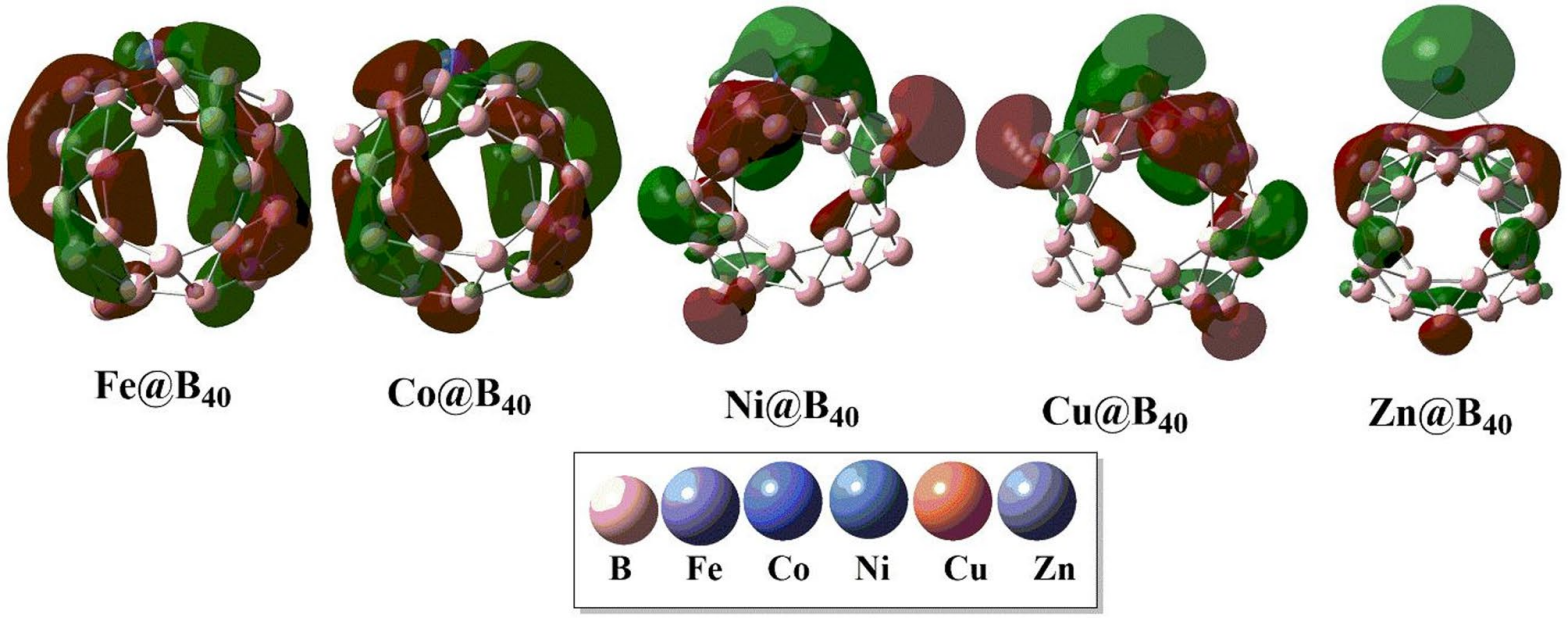
Isosurfaces of the main binding molecular orbitals of all TM@B_40_ complexes (TM = Fe, Co, Ni, Cu, Zn). All calculations are performed by using Gaussian 09 software, and results are visualized through visual builder’ GuassView 6.0 (https://gaussian.com/gaussview6/).

Our analytical approach proceeded in three stages: First, Mulliken partition analysis was performed to quantify orbital overlap between the TMs and B_40_ framework, identifying the principal binding molecular orbitals (M-orbs) through their percentage contributions (Tables S2-S6). The critical M-orbs were selected based on two criteria: (i) significant orbital overlap (15–85% contribution range) ensuring balanced participation from both TM and B_40_, and (ii) maximal overlap percentages among the first ten highest occupied molecular orbitals. Second, NAO analysis decomposed these primary M-orbs into their constituent atomic orbital contributions, with particular focus on TM orbital participation (Table 4). Finally, three-dimensional isosurface representations of the key M-orbs were generated to visualize the binding character (Fig. 8).

**Table 4:** The percentage contribution of TM to Main binding molecular orbital.

Complexes	% Contribution of TM	Orbital type
Fe	6.66	4*s*
	14.12	3*d*
Co	6.86	4*s*
	12.32	3*d*
Ni	8.86	4*s*
	10.90	3*d*
Cu	11.07	4*s*
	33.06	3*d*
Zn	29.87	4*s*
	0.79	3*d*

The analysis reveals distinct binding mechanisms across the TM series. For Fe@B_40_, Co@B_40_, Ni@B_40_, and Cu@B_40_ complexes, binding occurs predominantly through hybridization between TM 3d orbitals and boron 2p orbitals, consistent with chemisorption behavior. This is evidenced by the significant d-orbital contributions in the NAO decomposition (Table 4) and clearly visualized in the orbital isosurfaces (Fig. 8). The Zn@B_40_ complex exhibits markedly different behavior, with binding mediated primarily through the Zn 4s orbital rather than d-orbital participation. This increased s-character correlates with the longer interaction distance observed in Zn@B_40_, as discussed previously. Notably, the orbital analysis directly correlates with the interaction energy (E_int_) trends: The strong d-p hybridization in Fe-Co-Ni-Cu complexes corresponds to their substantial E_int_ values, while the weak s-type interaction in Zn@B_40_ explains its significantly lower binding energy. The absence of observable orbital overlap in Zn@B_40_ (Fig. 8) further confirms the physisorption nature of this complex, in contrast to the chemisorption character of the other TM@B_40_ systems.

These findings provide atomic-level insights into the bonding mechanisms governing TM-B_40_ interactions, establishing a direct connection between electronic structure and binding strength. The distinct hybridization patterns revealed here have important implications for tailoring the catalytic properties of boron-based nanocage systems through selective transition metal doping. The rest of the contribution to the main molecular orbital is done by 2p orbital of the Boron atom.

### HER catalytic efficiency of TM@B40 complexes

The Gibb’s free energy of hydrogen adsorption (ΔG_H_) was calculated to evaluate the hydrogen evolution reaction (HER) activity of the TM@B_40_ complexes. This thermodynamic parameter is crucial for assessing the catalytic efficiency of materials for HER, as it reflects the balance between hydrogen adsorption and desorption on the catalyst surface. An optimal ΔG_H_ value is close to zero, signifying a favorable adsorption–desorption equilibrium and thus excellent electrocatalytic performance^50^. Conversely, a highly positive ΔG_H_ suggests weak hydrogen adsorption, limiting the reaction kinetics, while a highly negative ΔG_H_ implies overly strong hydrogen binding, hindering H_2_ release and ultimately reducing catalytic efficiency.

**Fig. 9:**
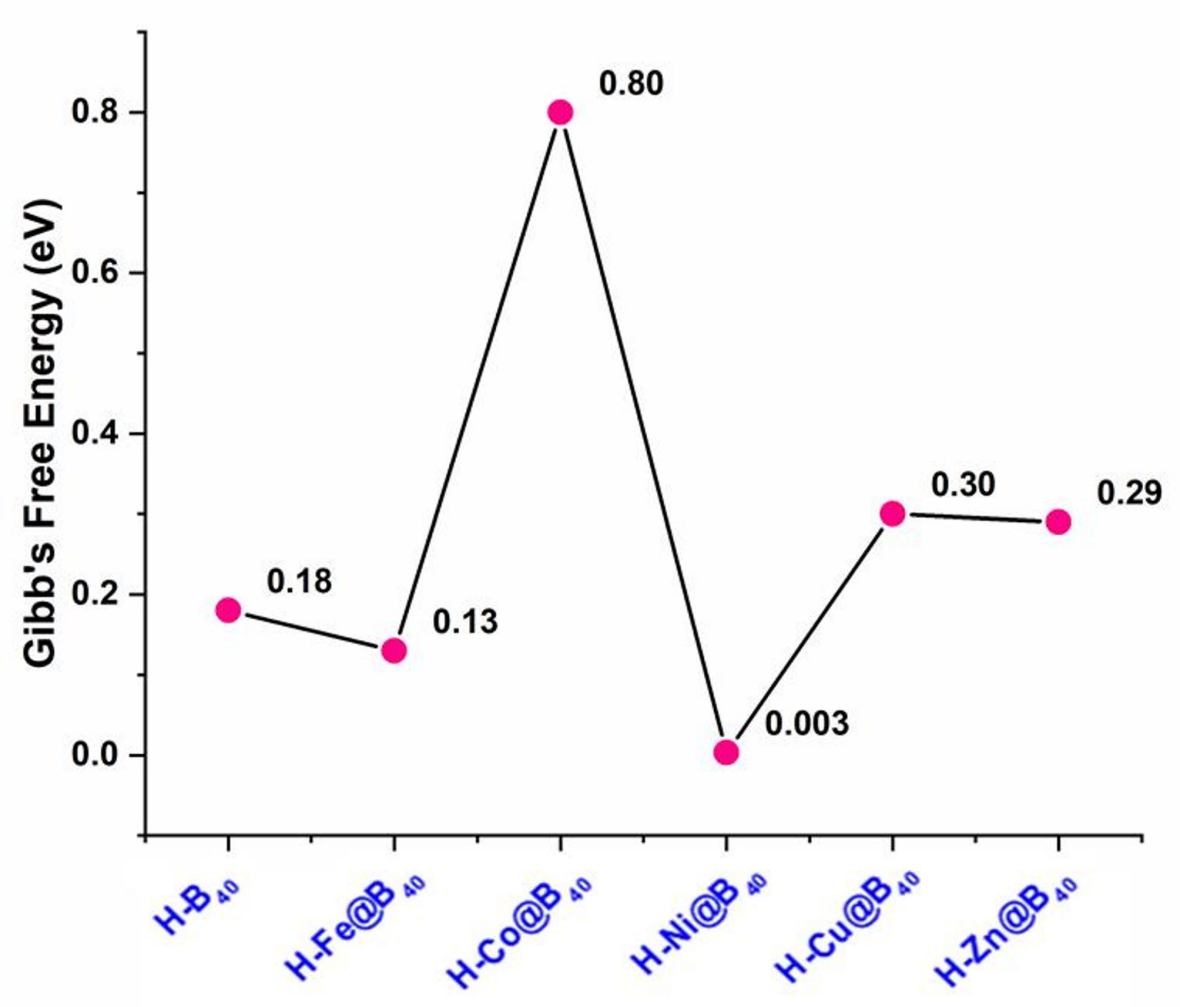
The Gibb’s free energy values (∆G_H*_) of hydrogen adsorbed TM@B_40_ SACs (TM = Fe, Co, Cu, Ni and Zn). The Gibb’s free energy values are in eV.

The calculated ΔG_H_ values for the designed TM@B_40_ complexes are summarized in Table 5 and graphically illustrated in Fig. 9. The calculated hydrogen adsorption free energies (ΔG_H_) for the TM@B_40_ complexes are presented in Table 5; Fig. 9. At the B3LYP/6-31G(d) level, the ΔG_H_ values are 0.13 eV (Fe@B_40_), 0.80 eV (Co@B_40_), 0.003 eV (Ni@B_40_), 0.30 eV (Cu@B_40_), and 0.29 eV (Zn@B_40_), compared to 0.18 eV for pristine B_40_. Notably, all complexes exhibit ΔG_H_ values within the optimal range for efficient HER activity, with Ni@B_40_ demonstrating particularly promising performance due to its near-thermoneutral ΔG_H_.

Inclusion of dispersion corrections (B3LYP-D3/6–31 + G(d)) reveals environment-dependent behavior. In the gas phase, ΔG_H_ values increase to 0.14 eV (H-B_40_), 0.52 eV (H-Fe@B_40_), 0.71 eV (H-Co@B_40_), -0.11 eV (H-Ni@B_40_), 0.17 eV (H-Cu@B_40_), and 0.39 eV (H-Zn@B_40_), showing a positive correlation with hydrogen adsorption energies (E_H*_). Remarkably, aqueous phase calculations yield distinct trends: while H-Fe@B_40_ (0.87 eV), H-Co@B_40_ (0.81 eV), and H-Zn@B_40_ (0.58 eV) show increased ΔG_H_, H-Ni@B_40_ (-0.01 eV) and H-Cu@B_40_ (0.01 eV) exhibit significantly improved performance approaching the ideal thermoneutral value. These results highlight two key findings: (1) Ni@B_40_ maintains superior HER activity across all computational methods and environments, and (2) Cu@B_40_ demonstrates dramatically enhanced performance in aqueous phase when dispersion corrections are included. This latter observation underscores the critical role of solvent effects and van der Waals interactions in stabilizing hydrogen adsorption intermediates.

The ΔG_H_ values of the best performers, H-Ni@B_40_ and H-Cu@B_40_, is also evaluated at the M06-L/def2-TZVP level of theory. The ΔG_H_ values in the gas phase are − 0.03 and 0.34 eV for H-Ni@B_40_ and H-Cu@B_40_, respectively. The same ΔG_H_ values are 0.05 and 0.23 eV for H-Ni@B_40_ and H-Cu@B_40_, respectively in aqueous phase. The ΔG_H_ values obtained at the M06-L/def2-TZVP level also show better results, which justify the superior HER activity of the Ni@B40 SAC.

**Table 5:** Comparison of interaction energy (E_int_ in eV), Deformation energy (E_def_ in eV), attraction energy (E_att_ in eV), adsorption energy of H interacting with TM@B_40_ (E_H_ in eV), Gibb’s free energy of H adsorption (∆G_H_ in eV) and counter-poise corrected energies (E_BSSE_ in eV) for our designed TM@B_40_ and H-TM@B_40_ complexes at B3LYP/6-31G (d) method in gas phase, B3LYP-D3/6–31 + G (d) method in gas phase and B3LYP-D3/6–31 + G (d) method in aqueous phase.

Pure and doped complexes	E_int_	E_def_	B3LYP-D3/6–31 + G (d) method in gas phase				B3LYP-D3/6–31 + G (d) method in aqueous phase			
			E_att_	E_H_	ΔG_H_	E_BSSE_	E_int_	E_H_	ΔG_H_	E_BSSE_
Fe@B_40_	-3.48	2.35	-5.83	-	-	-3.59	-3.72	-	-	-3.59
Co@B_40_	-3.51	0.33	-3.84	-	-	-3.65	-3.63	-	-	-3.65
Ni@B_40_	-2.64	2.22	-4.86	-	-	-2.83	-2.83	-	-	-2.83
Cu@B_40_	-1.88	0.38	-2.25	-	-	-2.05	-1.94	-	-	-2.05
Zn@B_40_	-0.36	0.09	-0.45	-	-	-0.31	-1.16	-	-	-0.32
H-B_40_	-	-	-	-0.07	0.14	-2.88	-	-0.08	0.14	-2.88
H-Fe@B_40_	-	-	-	0.29	0.52	-1.85	-	0.61	0.87	-1.86
H-Co@B_40_	-	-	-	0.45	0.71	-1.91	-	0.54	0.81	-1.91
H-Ni@ B_40_	-	-	-	-0.39	-0.11	-2.83	-	-0.29	-0.01	-2.83
H-Cu@B_40_	-	-	-	-0.07	0.17	-2.58	-	-0.25	0.01	-2.59
H-Zn@B_40_	-	-	-	0.20	0.39	-0.19	-	0.31	0.58	-0.39

Quantum theory of atoms in molecules (QTAIM), developed by Richard Bader^51^, was employed to analyze the nature of chemical interactions based on both quantitative and qualitative evaluations of electron density. The key concept is the bond critical pint (BCP), where properties such as values energy density, its Laplacian and energy density are used to characterize the nature of bonding or interaction. A higher electronic density at the BCP is indicative of covalent bonding, whereas significantly lower electron density is associated with non-covalent interactions, such as hydrogen bond or van der Waals forces. BCPs are identified between the B_40_ nanocage and transition metal atoms in TMs@B_40_ complexes, as well as between hydrogen and transition metals in H-TMs@B_40_ complexes. The density of all electrons (*ρ*) values at BCPs for all TMs@B_40_ and H-TMs@B_40_ complexes is between 0.01 and 0.17 au., suggesting an ionic character of the selected interactions. The Laplacian of electron density (∇^2^*ρ*) is positive for most complexes, indicating noncovalent bonding; however, negative ∇^2^*ρ* values observed for Zn@B_40_, H-B_40_ and H-Fe@B_40_ complexes confirm covalent character in these cases. The Lagrangian kinetic energy density [G(r)] at the CPs exceeds 35% for all complexes, consistent with non-covalent interactions, except for two CPs in Zn@B4_0_ complex, where values below 35% indicate covalent bonding. The potential energy density [V(r)] at the critical points is greater than [G(r)], further supporting the non-covalent nature of most interactions. The total energy density H(r) is negative for covalent, ionic or partially covalent interactions and positive for non-covalent interactions. Most CPs exhibit negative H(r) values, whereas a few CPs in Zn@B_40_ complex show positive values. Additionally, the -G(r)/V(r) ratio for the CPs in Zn@B_40_, H-B_40_ and Zn@B_40_ complexes is below 0.50, confirming covalent bonding while values below 0.10 for the remaining CPs indicate partially ionic or predominantly ionic interactions (See Table S7 and Figures S1-S2).

To further substantiate its catalytic efficiency, the ΔG_H_ of Ni@B_40_ was compared with those of the best-performing single-atom catalysts (SACs) reported in the literature (**see** Table 6). The comparison reveals that Ni@B_40_ exhibits competitive, if not superior, HER activity, underscoring its potential as an efficient SAC for hydrogen production. These findings are in agreement with earlier theoretical and experimental reports highlighting the electrocatalytic potential of transition metal-doped boron-based nanostructures^52^.

**Table 6:** Comparison of our designed Ni@B_40_ and Cu@B_40_ SACs with other reported transition metal-based SACs in literature.

Single atom Catalysts	∆G_H*_	References
MoS_2_/ZnO heterojunction	−0.04 eV	^53^
Ni/Cu-phthalocyanine/CNT catalysts	0.00 eV	^54^
Ru@8-CNB	0.12 eV	^55^
g-C_3_N_5_	-0.16 eV	^56^
Pt-doped VSeTe on Se side Pt-doped VSeTe on Te side	-0.02 eV 0.005 eV	^57^
Pt-Sb Alloy	-0.01	^58^
Pt@V-POM (0.02 eV)	0.02 eV	^59^
Pt-doped phenalenes	-0.24 eV	^60^
H-Ni@B_40_ H-Cu@B_40_	-0.01 0.01	This work

**Fig. 10:**
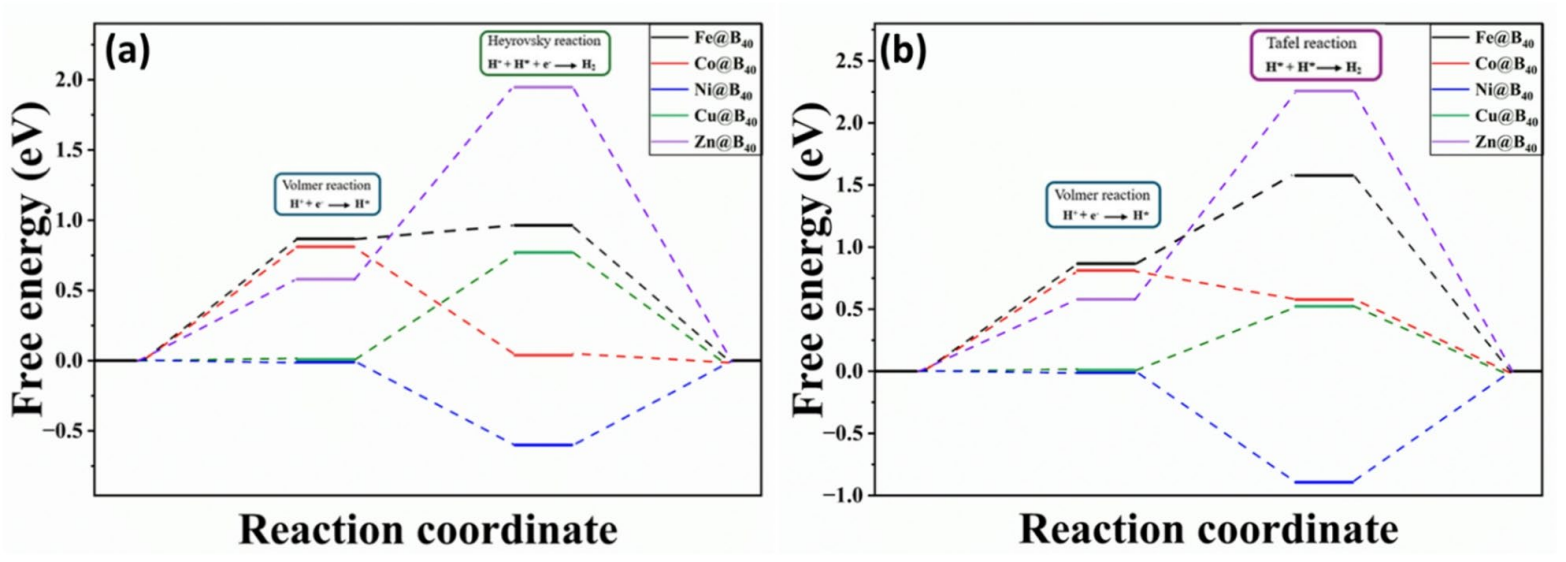
Volmer-Heyrovsky (**a**) and Volmer-Tafel (**b**) mechanistic pathways of hydrogen adsorbed TM@B_40_ SACs (TM = Fe, Co, Cu, Ni and Zn).

The HER is a critical half-reaction during electrochemical water splitting and proceeds through the reduction of protons to form molecular hydrogen via a series of coupled proton-electron transfer steps. According to the literature, HER in acidic media primarily follows two mechanistic pathways: Volmer-Heyrovsky and Volmer-Tafel mechanisms. The initial Volmer step involves proton adsorption and discharge on the catalytic surface, followed by either the electrochemical Heyrovsky reaction or the chemical Tafel recombination step. These reaction pathways are strongly dependent on the nature of the catalytic surface and the hydrogen binding energy. The Gibb’s free energy profiles diagrams for these processes are presented in Fig. 10. The thermal stability of these SACs is evaluated based on adsorption energies (E_ads_) at each reaction step^61–63^. During the Volmer step, the E_ads_ values of pure H-B_40_ and H-TM@B_40_ complexes ranges from − 0.29 to 0.61 eV, with the strongest adsorption (–0.29 eV) observed for H-Ni@B_40_ at B3LYP-D3/6–31 + G(d) method in aqueous phase. For the Heyrovsky step, the E_ads_ values range from − 0.88 to 1.68 eV, with peak values of − 0.88 eV for H-Ni@B_40_. In the Tafel step, the E_ads_ values range from − 1.18 to 1.99 eV, with the strongest adsorption (–1.18 eV) also found for H-Ni@B_40_. Across all three reaction steps, H-Ni@B_40_ complex consistently exhibits higher adsorption energies than the other SACs complexes, generally reflecting superior stability. These findings support the practical application of Ni@B_40_-SACs in HER catalysis.

The hydrogen adsorption free energy (ΔG_H*_) is a key descriptor of HER catalytic activity, where values close to zero signify optimal hydrogen binding and enhanced hydrogen evolution efficiency. The Gibb’s free energy values for each reaction step are summarized in Table 7. The investigated catalysts display a wide range of Gibb’s free energy (ΔG_H*_), ranging from − 0.60 to 1.95 eV in Volmer-Heyrovsky pathway, reflecting their varied hydrogen evolution performance. Among them, H-Ni@B_40_ (-0.01 eV) and H-C@B_40_ (0.01 eV) exhibit near-ideal ΔG_H*_ values. For the Volmer-Tafel pathway, ΔG_H*_ range from − 0.89 to 2.26 eV, with H-Ni@B_40_ again showing an optimal value (-0.01). Considering the individual reaction steps, H-Ni@B_40_ (-0.01 eV) and H-Cu@B_40_ (0.01 eV) display the lowest ΔG_H*_ values in the Volmer step, whereas H-Co@B_40_ (0.04) and H-Cu@B_40_ (0.52 eV) exhibit the most favorable ΔG_H*_ values in Heyrovsky and Tafel steps, respectively. In Volmer-Heyrovsky pathway, a lower barrier is observed for H-Ni@ B_40_ complex (0.60 eV), whereas in _the_ Volmer-Tafel pathway, the H-Cu@ B_40_ complex exhibits the lower barrier (0.52 eV). Overall, these results indicate facilitated hydrogen adsorption and desorption, leading to improved reaction kinetics and enhanced prospects for practical HER applications^64^.

**Table 7:** Adsorption energies (ΔE_ads_) and Gibb’s free energies (ΔG_H*_) of the Volmer, Heyrovsky, and Tafel steps for TM@B_40_ catalysts (in eV) at B3LYP-D3/6-31G (d) in aqueous phase

Catalysts	ΔE_ads_ (Volmer)	ΔG_H*_ (Volmer)	ΔE_ads_ (Heyrovsky)	ΔG_H*_ (Heyrovsky)	ΔE_ads_ (Tafel)	ΔG_H*_ (Tafel)
H-B_40_	-0.08	0.14	1.54	1.76	1.46	1.68
H-Fe@B_40_	0.61	0.87	0.71	0.96	1.32	1.58
H-Co@B_40_	0.54	0.81	-0.23	0.04	0.31	0.58
H-Ni@B_40_	-0.29	-0.01	-0.88	-0.60	-1.18	-0.89
H-Cu@B_40_	-0.25	0.01	0.51	0.77	0.27	0.52
H-Zn@B_40_	0.31	0.58	1.68	1.95	1.99	2.26

### Ab initio molecular dynamic (AIMD) simulations

**Fig. 11:**
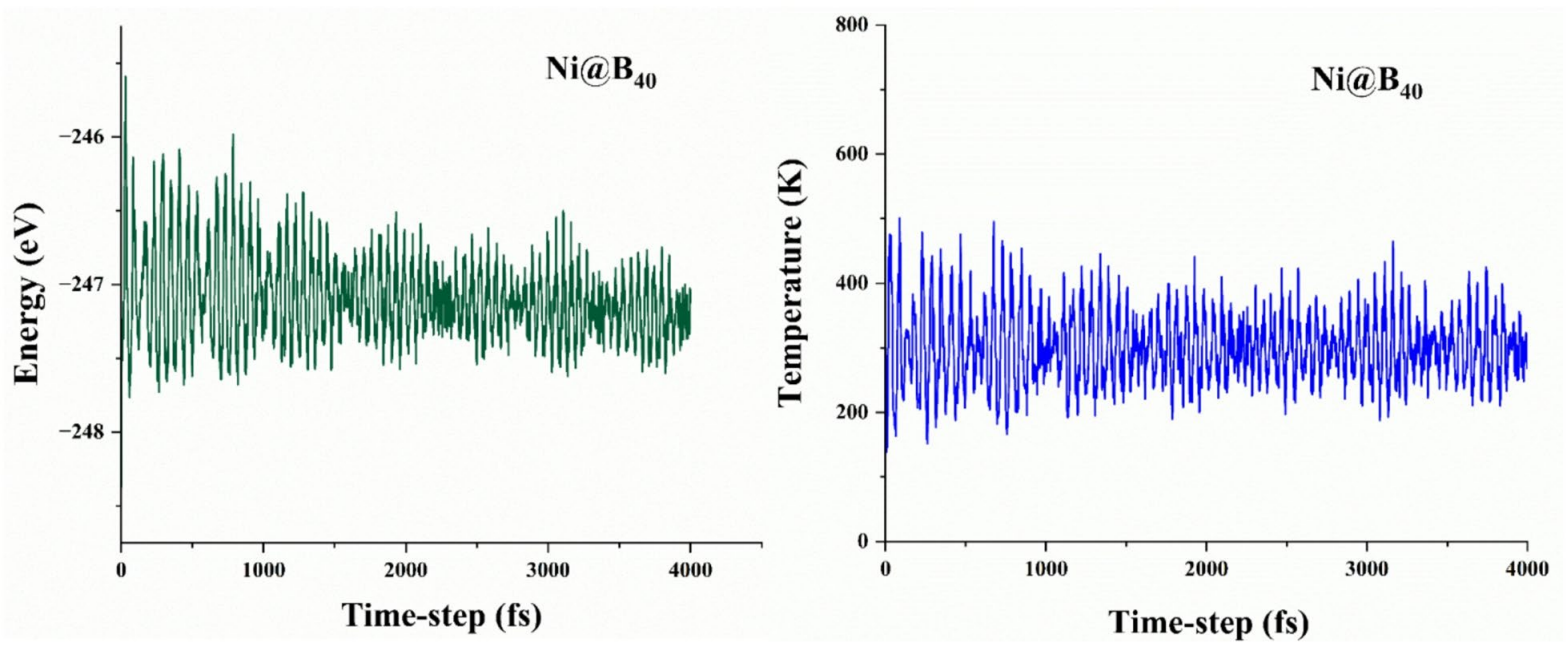
Time-dependent total energy (green, left) and temperature (blue, right) profiles for Ni@B_40_-SAC obtained from ab initio molecular dynamics simulations at 300 K over a 4000 time-steps femtosecond trajectory.

Ab initio molecular dynamic (AIMD) simulations are performed to evaluate the kinetic and thermal stability of the best-performing Ni@B4_0_ complex. All the simulations are conducted at 300 K over 4000 femtosecond time-steps. The thermal robustness and structural integrity of the complex are assessed through time- dependent temperature and total energy fluctuations, with the corresponding graphs presented in Fig. 11. The results indicate high stability for both temperature and total energy of Ni@B_40_ complex, as evidenced by the relatively narrow energy oscillations around a well-defined plateau (Fig. 11). The stable temperature profile demonstrates the ability of the Ni@B_40_ complex to maintain thermal equilibrium. These findings are consistent with the interaction energy results as discussed *vide supra*.

## Conclusion

This study presents a comprehensive theoretical investigation of late first-row transition metal-doped B_40_ nanocages (TM = Fe, Co, Ni, Cu, Zn) as single-atom catalysts for the hydrogen evolution reaction (HER). DFT and ab initio molecular dynamics (AIMD) calculations reveal that all TM@B_40_ complexes exhibit significant thermodynamic stability, with interaction energies (E_int_) ranging from − 1.16 to -3.72 eV at the B3LYP-D3/6–31 + G(d) level in aqueous phase, confirming their experimental viability. Notably, Ni@B_40_ and Cu@B_40_ demonstrate exceptional catalytic potential as single-atom catalysts, exhibiting near-thermoneutral hydrogen adsorption free energies (ΔG_H_) of -0.01 eV and 0.01 eV, respectively at the B3LYP-D3/6–31 + G(d) level in aqueous phase, which approach the optimal value for efficient HER activity. The enhanced catalytic performance stems from synergistic electronic and structural modifications. These systems have hydrogen adsorption energy values of -0.29 eV (H-Ni@B_40_) and − 0.25 eV (H-Cu@B_40_) at the B3LYP-D3/6–31 + G(d) level in aqueous phase, indicating favorable stabilization of reaction intermediates and also maintain their superior performance in aqueous environments. Natural Bond Orbital analysis reveals efficient charge delocalization upon Ni and Cu doping, while Density of States calculations demonstrate the emergence of new electronic states near the Fermi level upon hydrogen adsorption. These electronic modifications reduced HOMO-LUMO gaps of each complex and import conductive properties to them. These findings provide noble-metal-free HER catalysts and define key design principles for boron-based single-atom catalysts, providing a robust theoretical framework for future experimental work.

## Supplementary Information

Below is the link to the electronic supplementary material.

Supplementary Material 1.

## Data Availability

All data generated during and/or analyzed during the current study are available as part of the Main Text, the electronic Supplementary Material, or from the corresponding author upon reasonable request.
