# Supplementary material for: Breaking HER limits with Ni@B40’s single-atom catalytic prowess
Source: Sci Rep. 2026 Mar 31;16:15569. doi: 10.1038/s41598-026-46437-6 (PMC13187495; doi:10.1038/s41598-026-46437-6)
Supplement: Supplementary file 1 — Supplementary Material 1. [file 41598_2026_46437_MOESM1_ESM.docx]

**Supporting information**

**Breaking HER Limits with Ni@B_40_’s Single-Atom Catalytic Prowess**

Naveen Kosar^1,2^*, Saira Rafiq^3^, Sumayya M. Ansari^4^, Mona A. Aziz Aljar^5^, Muhammad Imran^6^, Ahmad Hasan^4^, Imran Malik^7^, Tariq Mahmood^5,8*^ and Adnan Younis^4^*

^1^Chemistry Department, King Fahd University of Petroleum & Minerals, Dhahran 31261, Saudi Arabia.

^2^IRC-RAC, King Fahd University of Petroleum & Minerals, Dhahran 31261, Saudi Arabia.

^3^Department of Chemistry, University of Management and Technology (UMT), C-11, Johar Town Lahore, Pakistan

^4^Department of Physics, United Arab Emirates University, P.O. Box 15551, Al Ain, United Arab Emirates

^5^Department of Chemistry, College of Science, University of Bahrain, Sakhir 32038, Bahrain

^6^Research Center for Advanced Materials Science (RCAMS), Chemistry Department, Faculty of Science, King Khalid University, P.O. Box 9004, Abha 61413, Saudi Arabia

^7^Department of Basic Sciences, Deanship of Preparatory Year and Supporting Studies, Imam Abdulrahman Bin Faisal University, P.O. Box 1982, Dammam 34212, Saudi Arabia

^8^Department of Chemistry, COMSATS University Islamabad, Abbottabad Campus, Abbottabad-22060, Pakistan

*To whom correspondence can be addressed: E-mail: [naveen.kosar@umt.edu.pk](mailto:naveen.kosar@umt.edu.pk) (N.K), [tmahmood@uob.edu.bh](mailto:tmahmood@uob.edu.bh) (T. M) and [adnaanyounis@yahoo.com](mailto:adnaanyounis@yahoo.com) (A. Y)

**Table S1.**  Interaction energy values (E_int_ in eV) of all complexes at PBE0-D3(BJ), M06, M06-L, ωB97X-D and B3LYP-D3 with def2-TZVP methods in gas phase and the E_int_ values in parenthesis represent the aqueous phase.

| **Complexes** | PBE0-D3(BJ)/def2-TZVP | M06/def2-TZVP | M06-L/def2-TZVP | B3LYP-D3/def2-TZVP |
| --- | --- | --- | --- | --- |
| **Fe@B_40_** | -6.25 | -7.07 | -8.13 | -6.31 |
| **Co@B_40_** | -3.56 | -2.03 | -5.54 | -3.77 |
| **Ni@B_40_** | -3.87 | -4.56 | -5.51 (-5.35) | -4.28 |
| **Cu@B_40_** | -1.74 | -2.27 | -3.22 (-3.01) | -2.04 |
| **Zn@B_40_** | -0.30 | -0.12 | -0.47 | -0.20 |
| **H-B_40_** | -0.02 | 0.04 | 0.43 | -0.02 |
| **H-Fe@B_40_** | 0.54 | 0.62 | 0.355 | 0.56 |
| **H-Co@B_40_** | 0.51 | -3.92 | -0.300 | 0.57 |
| **H-Ni@B_40_** | -0.34 | -0.37 | 0.09 (-0.29) | -0.44 |
| **H-Cu@B_40_** | -0.03 | 0.06 | -0.09 (0.10) | -0.11 |
| **H-Zn@B_40_** | 0.18 | 0.24 | 0.43 | -0.08 |

**Table S2.** Results of the Mulliken orbital composition analysis for Fe@B_40_.

| **Highest overlapping**  **orbitals A** | **Orbital index** | **Overlap %** | **Contribution %**  **from TM** |
| --- | --- | --- | --- |
| **1** | 67 | 5.61 | 5.33 |
| **2** | 703 | 5.06 | 4.40 |
| **3** | 96 | 4.89 | 16.52 |
| **4** | 738 | 4.63 | 21.72 |
| **5** | 731 | 4.52 | 7.54 |
| **6** | 739 | 4.46 | 21.13 |
| **7** | 70 | 4.38 | 5.35 |
| **8** | 95 | 4.36 | 24.98 |
| **9** | 69 | 4.28 | 4.99 |
| **10** | 85 | 4.12 | 15.58 |

**Table S3.** Results of the Mulliken orbital composition analysis for Co@B_40_.

| **Highest overlapping**  **orbitals A** | **Orbital index** | **Overlap %** | **Contribution %**  **from TM** |
| --- | --- | --- | --- |
| **1** | 68 | 6.07 | 1.07 |
| **2** | 704 | 5.89 | 1.04 |
| **3** | 71 | 5.86 | 2.36 |
| **4** | 96 | 5.44 | 14.09 |
| **5** | 707 | 5.39 | 1.60 |
| **6** | 752 | 5.21 | 2.47 |
| **7** | 70 | 4.96 | 5.85 |
| **SF8** | 97 | 4.82 | 0.98 |
| **9** | 706 | 4.71 | 4.71 |
| **10** | 116 | 4.62 | 3.73 |

**Table S4.** Results of the Mulliken orbital composition analysis for Ni@B_40_.

| **Highest overlapping**  **orbitals A** | **Orbital index** | **Overlap %** | **Contribution %**  **from TM** |
| --- | --- | --- | --- |
| **1** | 703 | 6.59 | 5.83 |
| **2** | 67 | 6.56 | 5.89 |
| **3** | 70 | 6.55 | 7.69 |
| **4** | 706 | 6.47 | 5.73 |
| **5** | 751 | 5.98 | 13.12 |
| **6** | 69 | 5.37 | 5.90 |
| **7** | 705 | 5.14 | 5.11 |
| **8** | 708 | 5.05 | 4.87 |
| **9** | 72 | 4.99 | 4.98 |
| **10** | 74 | 4.21 | 8.41 |

**Table S5.** Results of the Mulliken orbital composition analysis for Cu@B_40_.

| **Highest overlapping**  **orbitals A** | **Orbital index** | **Overlap %** | **Contribution %**  **from TM** |
| --- | --- | --- | --- |
| **1** | 703 | 8.00 | 7.09 |
| **2** | 67 | 7.83 | 6.98 |
| **3** | 706 | 6.77 | 7.96 |
| **4** | 70 | 6.67 | 8.04 |
| **5** | 716 | 5.92 | 32.16 |
| **6** | 80 | 5.75 | 32.55 |
| **7** | 707 | 5.57 | 6.77 |
| **8** | 71 | 5.49 | 6.49 |
| **9** | 705 | 5.43 | 5.73 |
| **10** | 69 | 5.36 | 5.73 |

**Table S6.** Results of the Mulliken orbital composition analysis for Zn@B_40_.

| **Highest overlapping**  **orbitals A** | **Orbital index** | **Overlap %** | **Contribution %**  **from TM** |
| --- | --- | --- | --- |
| **1** | 163 | 5.76 | 32.24 |
| **2** | 69 | 4.42 | 8.31 |
| **3** | 116 | 4.23 | 6.88 |
| **4** | 100 | 3.33 | 3.34 |
| **5** | 166 | 3.09 | 32.98 |
| **6** | 66 | 3.05 | 2.59 |
| **7** | 103 | 2.61 | 3.63 |
| **8** | 108 | 2.32 | 2.03 |
| **9** | 90 | 2.29 | 3.30 |
| **10** | 122 | 2.21 | 7.84 |

**Table S7.** The density of all electrons (ρ), Laplacian of electron density (∇^2^ρ), Lagrangian kinetic energy [G(r)], Potential energy density [V(r)], Total energy density (H(r)) and their ratio [-G(r)/V(r)] for selected critical points of TMs@B_40_, H-B40 and H-TMs@B_40_ complexes.

| **Complexes** | **CPs** | ***ρ*** | **∇^2^*ρ*** | **G(r)** | **V(r)** | **H(r)** | **-G(r)/V(r)** |
| --- | --- | --- | --- | --- | --- | --- | --- |
| [**Co@B_40_**](mailto:Co@B40) | 50 | 0.09 | 0.09 | 0.06 | -0.10 | -0.04 | 0.61 |
|  | 52 | 0.09 | 0.06 | 0.06 | -0.11 | -0.05 | 0.57 |
|  | 54 | 0.09 | 0.06 | 0.06 | -0.11 | -0.05 | 0.57 |
|  | 57 | 0.10 | 0.08 | 0.07 | -0.12 | -0.05 | 0.58 |
|  | 59 | 0.10 | 0.08 | 0.07 | -0.12 | -0.05 | 0.58 |
| **Fe@B40** | 74 | 0.09 | 0.11 | 0.07 | -0.11 | -0.04 | 0.62 |
|  | 81 | 0.09 | 0.11 | 0.07 | -0.11 | -0.04 | 0.62 |
|  | 86 | 0.09 | 0.07 | 0.06 | -0.10 | -0.04 | 0.59 |
|  | 89 | 0.09 | 0.07 | 0.06 | -0.10 | -0.04 | 0.59 |
|  | 94 | 0.08 | 0.12 | 0.06 | -0.10 | -0.04 | 0.59 |
| [**Ni@B_40_**](mailto:Ni@B40) | 44 | 0.09 | 0.07 | 0.06 | -0.10 | -0.04 | 0.58 |
|  | 47 | 0.08 | 0.07 | 0.05 | -0.09 | -0.03 | 0.60 |
|  | 50 | 0.08 | 0.07 | 0.05 | -0.09 | -0.03 | 0.60 |
|  | 52 | 0.09 | 0.06 | 0.06 | -0.11 | -0.05 | 0.57 |
|  | 54 | 0.09 | 0.06 | 0.06 | -0.11 | -0.05 | 0.57 |
| [**Cu@B_40_**](mailto:Cu@B40) | 46 | 0.07 | 0.10 | 0.05 | -0.08 | -0.03 | 0.66 |
|  | 51 | 0.08 | 0.07 | 0.06 | -0.10 | -0.04 | 0.59 |
|  | 53 | 0.08 | 0.07 | 0.06 | -0.10 | -0.04 | 0.59 |
| [**Zn@B_40_**](mailto:Zn@B40) | 51 | 0.01 | 0.02 | 0.00 | 0.00 | 0.00 | 1.15 |
|  | 54 | 0.14 | -0.16 | 0.06 | -0.17 | -0.10 | 0.38 |
|  | 56 | 0.01 | 0.02 | 0.00 | 0.00 | 0.00 | 1.15 |
| **H-B_40_** | 48 | 0.17 | -0.32 | 0.10 | -0.28 | -0.18 | 0.36 |
| [**Fe@B_40_**](mailto:Fe@B40) | 55 | 0.17 | -0.31 | 0.10 | -0.28 | -0.18 | 0.36 |
| [**Co@B_40_**](mailto:Co@B40) | 51 | 0.14 | 0.08 | 0.09 | -0.16 | -0.07 | 0.56 |
| [**Ni@B_40_**](mailto:Ni@B40) | 51 | 0.12 | 0.05 | 0.08 | -0.15 | -0.07 | 0.54 |
| [**Cu@B_40_**](mailto:Cu@B40) | 51 | 0.11 | 0.06 | 0.08 | -0.14 | -0.06 | 0.56 |
| [**Zn@B_40_**](mailto:Zn@B40) | 47 | 0.11 | 0.07 | 0.08 | -0.14 | -0.06 | 0.56 |





**Figure S1:** QTAIM analysis figures representing selected critical points of TMs@B_40_, complexes.





**Figure S2:** QTAIM analysis figures representing selected critical points of H-B40 and H-TMs@B_40_ complexes.

**Table S8.** Cartesian coordinates, the first frequencies (without any imaginary frequencies because all are stable structures not unstable transition states), and computed total energies of optimized pure, transition metal doped B_40_ and hydrogen adsorbed TM@B_40_ complexes, along with any other absolute energy values.

| **S. No.** | **Cartesian coordinates** | **First frequency (Hertz)** | **Total energies (Hartree)** | **Absolute energies (Hartree)** |
| --- | --- | --- | --- | --- |
| 1. | B_40_  B 0.38549700 -2.74161400 -1.75822700  B -2.85617000 -0.00122500 -1.69829700  B -1.20085400 -2.37219100 -1.38435300  B 0.38549700 2.74162000 1.75824600  B 2.85617400 1.69831400 -0.00121200  B 1.99013100 -0.00194200 -2.63794100  B -1.67551300 0.88237200 2.37906900  B 1.20064700 -1.38494500 2.37163200  B 0.38547100 -2.73902900 1.76224800  B -2.85620000 0.00125700 1.69824400  B -1.20088600 -2.37016500 1.38782200  B -0.38526500 1.75823700 -2.74171400  B -1.20081100 2.37018500 -1.38780700  B -1.20083500 2.37222200 1.38429300  B 1.67576200 2.38176100 0.87874700  B 1.67575300 -2.38177900 -0.87871300  B -1.67551100 -0.87888300 2.38033900  B 1.67578100 2.38046700 -0.88220000  B -1.99125900 2.63893000 -0.00195500  B -0.38528900 -1.76227000 -2.73914800  B -1.67547100 -0.88236800 -2.37908100  B -1.67546100 0.87888300 -2.38038700  B 1.20065800 1.38841900 2.36958800  B 1.20069300 -1.38842600 -2.36957800  B 1.20071200 1.38494100 -2.37159900  B -2.53297500 1.39064800 0.89206800  B -2.53296600 1.38934400 -0.89415400  B 0.38553600 2.73903100 -1.76225800  B 2.53328900 0.89174000 -1.39047700  B 1.99007700 0.00192500 2.63798100  B 1.67574500 -2.38049800 0.88222800  B -2.53300600 -1.38931500 0.89409800  B -2.53298800 -1.39062300 -0.89210800  B 2.53328300 -0.89380700 -1.38918200  B -0.38532900 1.76226600 2.73912500  B -1.99125100 -2.63889800 0.00191700  B 2.53326500 0.89377400 1.38922200  B 2.85615400 -1.69834100 0.00126800  B -0.38533200 -1.75824800 2.74172300  B 2.53324800 -0.89176700 1.39053100 | 172.89 | -993.741729 | -993.566665 |
| 2. | Fe@B_40_  B -2.41053800 -0.40604800 -1.89744800  B 0.35808000 2.83071200 -1.66127600  B -2.07554700 1.18623900 -1.44358600  B 3.14292200 -0.37324600 1.75818400  B 2.09268800 -2.82753000 -0.00483900  B 0.38822900 -1.94119900 -2.62067000  B 1.28967500 1.67705800 2.36250700  B -1.06440600 -1.24221600 2.39501200  B -2.41183900 -0.41320900 1.89433300  B 0.35697500 2.82458800 1.67266100  B -2.07661200 1.18063400 1.44784700  B 2.15961400 0.39709600 -2.73963300  B 2.76874700 1.23722500 -1.40458300  B 2.76826000 1.23158800 1.41067300  B 2.79284200 -1.67013300 0.88987600  B -2.00598600 -1.67410500 -0.87800600  B -0.45085800 1.64684100 2.41643700  B 2.79298300 -1.66655900 -0.89456200  B 3.02516800 1.99667600 0.00472300  B -1.35085400 0.38678300 -2.78053900  B -0.44923200 1.65625700 -2.41076600  B 1.29161300 1.68637200 -2.35554200  B 1.79923000 -1.20304700 2.38778900  B -1.06229800 -1.23233000 -2.40036400  B 1.80090000 -1.19355300 -2.39176000  B 1.77508600 2.55048800 0.89307900  B 1.77614200 2.55452600 -0.88254500  B 3.14388500 -0.36623000 -1.75753200  B 1.31485800 -2.51320300 -1.41178100  B 0.38659300 -1.95132300 2.61303300  B -2.00581700 -1.67782500 0.87067800  B -1.05869200 2.48478700 0.91607600  B -1.05824800 2.48795300 -0.90752000  B -0.46741200 -2.52412300 -1.37777400  B 2.15769300 0.38640100 2.74179400  B -2.31129700 1.98319800 0.00312300  B 1.31392900 -2.51922100 1.40261800  B -1.28087300 -2.86549300 -0.00627500  B -1.35192100 0.37551400 2.78113200  B -0.46873800 -2.52913600 1.36752800  Fe -2.94902700 0.00553700 -0.00040500 | 157.06 | -2257.353545 | -2257.177906 |
| 3. | Co@B_40_  B -2.42099800 -0.38756300 -1.89313500  B 0.36856000 2.83081200 -1.67321100  B -2.05666600 1.20051100 -1.45139900  B 3.13852500 -0.38674200 1.76497900  B 2.07738600 -2.83313500 -0.00869000  B 0.37679900 -1.94552400 -2.62931100  B 1.29405800 1.67279500 2.37873000  B -1.06118600 -1.22332400 2.39371100  B -2.42070300 -0.39942100 1.88989600  B 0.36811800 2.82054800 1.69093500  B -2.05724000 1.19152600 1.45871000  B 2.16231700 0.39582000 -2.74916700  B 2.76658900 1.22236200 -1.40195200  B 2.76621700 1.21370300 1.41000000  B 2.77504800 -1.67408500 0.88580000  B -2.00134700 -1.65282000 -0.88216200  B -0.45621400 1.63175000 2.41733200  B 2.77549500 -1.66857600 -0.89574300  B 3.02152400 1.98060000 0.00648800  B -1.36896900 0.39175500 -2.80192100  B -0.45594700 1.64669300 -2.40682400  B 1.29488200 1.68770400 -2.36805700  B 1.78439800 -1.20540400 2.38596900  B -1.06029200 -1.20783800 -2.40106000  B 1.78521900 -1.19053600 -2.39331800  B 1.77323900 2.53284300 0.89792400  B 1.77354100 2.53826700 -0.88186100  B 3.13925600 -0.37583000 -1.76704500  B 1.29472300 -2.51120100 -1.41063200  B 0.37623400 -1.96221700 2.61717900  B -2.00141800 -1.65846800 0.87158200  B -1.03882800 2.48794400 0.93071300  B -1.03860000 2.49366600 -0.91561300  B -0.48314300 -2.52051700 -1.38750400  B 2.16176300 0.37872100 2.75179000  B -2.27851600 1.98262400 0.00584100  B 1.29459200 -2.52017800 1.39518900  B -1.29397300 -2.85566500 -0.00893900  B -1.36898400 0.37404200 2.80401500  B -0.48340000 -2.52994900 1.37192800  Co -2.81890000 0.00635300 -0.00021600 | 149.13 | -2376.443486 | -2376.267472 |
| 4. | Ni@B_40_  B -2.41843600 -0.41670500 -1.89796800  B 0.38229200 2.84022200 -1.69138300  B -2.00138800 1.19985200 -1.48261000  B 3.15412800 -0.37500100 1.76692200  B 2.11636600 -2.84128700 0.00216600  B 0.39087100 -1.96099900 -2.60408700  B 1.29602400 1.69263500 2.37509300  B -1.03076400 -1.20550600 2.36182300  B -2.41802700 -0.41364800 1.89820600  B 0.38163700 2.84190700 1.68706600  B -2.00169900 1.20150500 1.48001700  B 2.16867100 0.39482500 -2.73947100  B 2.78242800 1.21327800 -1.38978500  B 2.78210200 1.21518000 1.38851700  B 2.79641300 -1.66704300 0.89131000  B -1.97232700 -1.64398400 -0.87033600  B -0.47358800 1.63389500 2.39273500  B 2.79544300 -1.66766500 -0.88879800  B 3.04708400 1.99254900 -0.00123000  B -1.36781800 0.38355400 -2.81287800  B -0.47381600 1.63036200 -2.39311000  B 1.29639100 1.69052100 -2.37880500  B 1.79410000 -1.18706100 2.38148500  B -1.03124300 -1.20967900 -2.36013600  B 1.79430800 -1.19060100 -2.37986800  B 1.76917100 2.52550700 0.87809000  B 1.76933500 2.52418000 -0.88146400  B 3.15404200 -0.37720100 -1.76648300  B 1.31060100 -2.51495600 -1.38233600  B 0.39083500 -1.95720900 2.60705500  B -1.97403700 -1.64356200 0.87284400  B -1.01343100 2.49955900 0.91856100  B -1.01248800 2.49822600 -0.92179100  B -0.46412100 -2.54886300 -1.35856600  B 2.16896700 0.39829300 2.73920300  B -2.24819200 1.95408700 -0.00140300  B 1.31032300 -2.51310000 1.38597800  B -1.29297700 -2.90271700 0.00197200  B -1.36910100 0.38823100 2.81317800  B -0.46399700 -2.54515300 1.36165300  Ni -2.82572900 0.01135200 -0.00024400 | 120.31 | -2501.933962 | -2501.760135 |
| 5. | Cu@B_40_  B -2.38663300 -0.41916000 -1.88289900  B 0.40349900 2.84699700 -1.68363500  B -1.97408700 1.21438900 -1.47075800  B 3.17729800 -0.37566200 1.76227900  B 2.14957700 -2.84516200 -0.00383400  B 0.41756600 -1.96050100 -2.60368000  B 1.30980900 1.68743000 2.37276300  B -0.99463200 -1.21117700 2.34453700  B -2.38537900 -0.42364900 1.88300900  B 0.40454800 2.84220300 1.69047700  B -1.97476800 1.21089900 1.47387200  B 2.18153300 0.40105300 -2.72835000  B 2.80617800 1.21761200 -1.38035800  B 2.80715200 1.21373700 1.38127300  B 2.82870100 -1.66967900 0.88379000  B -1.94721200 -1.67502900 -0.87821800  B -0.45296600 1.64298900 2.39744500  B 2.82728400 -1.66686400 -0.88901600  B 3.07437900 2.00748700 0.00145000  B -1.34474200 0.39210000 -2.79405200  B -0.45408700 1.64761800 -2.39200900  B 1.30812600 1.69442400 -2.37008700  B 1.81576200 -1.19025100 2.36706600  B -0.99574200 -1.20565300 -2.34630500  B 1.81385300 -1.18466600 -2.37084500  B 1.79295400 2.52705200 0.88163700  B 1.79215000 2.52921600 -0.87637500  B 3.17544500 -0.37097100 -1.76463700  B 1.33798500 -2.51455200 -1.38397900  B 0.41926600 -1.96559800 2.59920200  B -1.94692200 -1.67761000 0.87556100  B -0.99296900 2.50425300 0.90809200  B -0.99454100 2.50849100 -0.90204300  B -0.44092100 -2.54761800 -1.35614700  B 2.18409100 0.39424600 2.72860100  B -2.23516400 1.97970300 0.00260200  B 1.33878200 -2.51759300 1.37782700  B -1.28495500 -2.91532400 -0.00305500  B -1.34309000 0.38559400 2.79500600  B -0.43949800 -2.54910500 1.34973900  Cu -2.89269500 0.00660800 0.00069900 | 121.33 | -2634.082465 | -2633.908070 |
| 6. | Zn@B_40_  B -0.21926700 1.74105000 -2.73974100  B -3.46496300 1.69624600 -0.00181600  B -1.81460500 1.38646400 -2.38546800  B -0.21937900 -1.74114800 2.73974600  B 2.28770400 0.00170700 1.65122700  B 1.35234600 2.66005400 -0.00281600  B -2.27675800 -2.37362100 0.88136000  B 0.58772200 -2.37265400 -1.39507500  B -0.21944400 -1.74692100 -2.73602500  B -3.46508300 -1.69605200 0.00180600  B -1.81473600 -1.39152000 -2.38253100  B -1.00064800 2.73699800 1.77067300  B -1.81466400 1.39155700 2.38263300  B -1.81461200 -1.38633300 2.38532500  B 1.07751900 -0.86463700 2.35954100  B 1.07772300 0.86462700 -2.35965200  B -2.27697700 -2.37558100 -0.87643600  B 1.07758800 0.86959100 2.35781100  B -2.60121500 0.00284700 2.64134700  B -1.00057500 2.73320600 -1.77642300  B -2.27669100 2.37373600 -0.88145000  B -2.27674400 2.37561300 0.87641300  B 0.58774200 -2.36986900 1.40011200  B 0.58790000 2.36980100 -1.40020800  B 0.58777200 2.37267200 1.39506900  B -3.14175900 -0.88684600 1.38672600  B -3.14153300 0.88985600 1.38469100  B -0.21930700 1.74689900 2.73606800  B 1.89243500 1.39730600 0.87049900  B 1.35232600 -2.66014200 0.00281500  B 1.07737500 -0.86957100 -2.35759700  B -3.14172000 -0.88978200 -1.38482000  B -3.14157200 0.88691400 -1.38663100  B 1.89237800 1.39538500 -0.87338900  B -1.00074100 -2.73324700 1.77642500  B -2.60118500 -0.00276000 -2.64134700  B 1.89233600 -1.39549300 0.87346800  B 2.28773000 -0.00180500 -1.65112900  B -1.00075600 -2.73695300 -1.77053700  B 1.89245600 -1.39740800 -0.87048400  Zn 4.07231400 -0.00003100 -0.00003000 | 83.13 | -2772.828112 | -2772.655910 |
| 7. | H@B_40_  B 2.74863400 0.65127400 -1.71733400  B 0.39883900 3.20890800 0.71549300  B 1.70841400 1.94755400 -1.55660100  B -2.42662900 -1.34746300 1.71683300  B 0.38710000 -2.88222300 1.54906100  B 2.97952200 -0.96353300 1.14694700  B -2.83954700 0.60268700 -0.22218700  B -0.96883800 -1.67865000 -2.28022900  B -0.20588700 -0.51788900 -3.25013700  B -2.42134200 2.08646000 -0.74725700  B -0.61436500 1.02320200 -2.76383600  B 1.49627000 1.01194600 2.79081400  B -0.18469200 1.22266900 2.75782300  B -2.62357100 0.30023700 1.52554400  B -1.05984600 -2.14541000 1.76388100  B 2.33446200 -0.89603800 -1.76548500  B -2.24292100 0.88999900 -1.82951900  B 0.43559300 -1.61832700 2.55172900  B -1.74649700 1.39168000 2.35777400  B 2.89459200 1.54887100 -0.41445200  B 1.76661500 2.48864500 0.21893000  B 1.05365900 2.21287100 1.82043100  B -2.09239900 -2.08571300 0.23960400  B 3.01477300 -0.10744500 -0.22279400  B 1.92216700 -0.52656300 2.29198300  B -2.21090800 1.81755100 0.85444500  B -0.69707500 2.42759500 1.64108100  B 0.56262400 -0.18554900 3.24953600  B 1.75953400 -2.01098400 1.41676900  B -1.47313300 -2.69249200 -1.12580700  B 0.85628400 -1.47492500 -2.52376700  B -1.08014000 2.27122400 -1.66318000  B 0.40333800 2.85491900 -0.88500100  B 2.47315900 -1.74477300 -0.19188100  B -3.37203300 -0.89179700 0.39406300  B 0.36404200 2.25886400 -2.40309500  B -0.57135900 -2.88488100 0.21714800  B 1.74749700 -2.38744200 -1.51705700  B -1.70546700 -0.27283000 -2.79166800  B 0.13626100 -2.67108800 -1.39792200  H -4.53365800 -1.15571600 0.24660300 | 169.46 | -994.330811 | -994.148379 |
| 8. | H-Fe@B_40_  B 2.30666300 1.52382300 1.37324800  B -0.51441700 2.90713900 -1.50661700  B 1.95592700 1.98741600 -0.21786100  B -3.08407300 -1.83844100 -0.65374100  B -2.04103900 -1.60632400 2.37941800  B -0.48012000 1.16970500 3.05754200  B -1.24595300 -1.15643900 -2.70616900  B 1.21145800 -2.61439100 -0.22397100  B 2.73729400 -1.79032000 -0.70120600  B -0.36379500 0.07811600 -3.25282400  B 2.10177300 -0.49582800 -1.75384900  B -2.30989200 2.42838200 1.11978300  B -2.86746300 1.69850200 -0.30552400  B -2.77797000 -0.66140500 -1.81264500  B -2.70794500 -1.76462300 0.90283500  B 1.99703800 -0.03220400 1.90805500  B 0.50753800 -1.12357500 -2.57551300  B -2.80828800 -0.26920500 1.87598800  B -3.09783100 0.92299700 -1.70828800  B 1.18744000 2.64316600 1.17585700  B 0.29222300 2.96049800 -0.11332300  B -1.44439800 2.82731500 -0.16707800  B -1.69247500 -2.73027900 -0.27298500  B 0.95252000 1.44955000 2.33520800  B -1.88592200 1.30186000 2.27292900  B -1.79699300 0.52217100 -2.61659000  B -1.89267700 2.02451700 -1.69323500  B -3.22752200 1.13919800 1.24218500  B -1.31968700 -0.21395300 2.85200000  B -0.22932400 -3.22337400 0.22455900  B 2.01193300 -1.46541200 0.92911200  B 1.02104500 0.61312400 -2.55651100  B 0.94637100 2.16432500 -1.61997800  B 0.45041600 -0.14022400 2.90860000  B -2.06178200 -2.19945900 -1.81054700  B 2.24791300 1.17501500 -1.64679200  B -1.20709200 -2.57518300 1.36425700  B 1.32575000 -1.43200700 2.44078200  B 1.48975900 -2.08825200 -1.80279100  B 0.61616300 -2.45593600 1.37728900  Fe 2.87256000 0.15591000 0.02425800  H 3.80061500 -2.35358700 -0.73875800 | 145.57 | -2257.978158 | -2257.795332 |
| 9. | H-Co@B_40_  B -2.40928900 0.30414200 1.89626100  B 0.47767500 -2.81810500 1.68945100  B -1.99523600 -1.25982000 1.46019800  B 3.14528100 0.47001600 -1.76824000  B 2.00283600 2.88306200 -0.00559900  B 0.33153000 1.95194700 2.61561100  B 1.36819100 -1.64520800 -2.36872900  B -1.09032000 1.16899600 -2.39922000  B -2.40940500 0.29683000 -1.89708500  B 0.47751300 -2.82453000 -1.67868200  B -1.99553200 -1.26545600 -1.45518100  B 2.19686400 -0.31986400 2.75537100  B 2.81792300 -1.13632600 1.41190500  B 2.81787100 -1.14176000 -1.40784200  B 2.74704200 1.75275100 -0.89554900  B -2.03194900 1.58231500 0.87186600  B -0.38519100 -1.67960200 -2.42673600  B 2.74715400 1.75619000 0.88860900  B 3.09366700 -1.89008600 0.00347300  B -1.33373300 -0.43767500 2.80764900  B -0.38496300 -1.67030400 2.43318200  B 1.36837500 -1.63607400 2.37486500  B 1.76082000 1.23945400 -2.39363000  B -1.09003400 1.17819400 2.39486900  B 1.76113800 1.24862100 2.38874600  B 1.87483400 -2.49564800 -0.88928700  B 1.87494500 -2.49226400 0.89867000  B 3.14546900 0.47676900 1.76611100  B 1.23774000 2.54665700 1.40533700  B 0.33122700 1.94198100 -2.62302600  B -2.03165700 1.57882500 -0.87745300  B -0.92695700 -2.51751900 -0.90790200  B -0.92686900 -2.51409900 0.91761000  B -0.53685100 2.48949400 1.35706200  B 2.19657800 -0.33041400 -2.75431200  B -2.18714500 -2.03578800 0.00403700  B 1.23759000 2.54129700 -1.41517100  B -1.37986100 2.80782100 -0.00519800  B -1.33404700 -0.44846500 -2.80580200  B -0.53699400 2.48422500 -1.36634700  Co -2.81739400 -0.05252500 0.00013200  H -4.06152000 0.71527800 -0.00301500 | 97.37 | -2634.668041 | -2634.486314 |
| 10 | H-Ni@B_40_  B 2.37105800 0.36457200 -1.86441200  B -0.45276500 -2.82959000 -1.68350100  B 1.97136400 -1.22432700 -1.44658800  B -3.18283400 0.41358100 1.76534900  B -2.10255300 2.85574500 -0.00160700  B -0.40783400 1.96189400 -2.62254100  B -1.35452000 -1.66664500 2.37807800  B 1.00689700 1.19076700 2.37296100  B 2.37093500 0.36662000 1.86438800  B -0.45290900 -2.82775400 1.68646500  B 1.97120400 -1.22267900 1.44804400  B -2.21094300 -0.36911200 -2.74733700  B -2.82631000 -1.18622200 -1.39903400  B -2.82643600 -1.18473100 1.40001600  B -2.81341600 1.69810800 0.88425300  B 1.96565400 1.64253700 -0.87727600  B 0.40399500 -1.65501300 2.40446000  B -2.81327700 1.69711500 -0.88628900  B -3.09376800 -1.95444000 0.00088900  B 1.32593800 -0.40565000 -2.78560200  B 0.40424500 -1.65760400 -2.40262400  B -1.35429300 -1.66922600 -2.37644000  B -1.81508900 1.21282700 2.38373400  B 1.00715000 1.18824100 -2.37404200  B -1.81477100 1.21030300 -2.38515400  B -1.85054900 -2.51808300 0.89161400  B -1.85045500 -2.51902800 -0.88911600  B -3.18262900 0.41170300 -1.76611000  B -1.31964000 2.53020700 -1.40370900  B -0.40812300 1.96457600 2.62046400  B 1.96595300 1.64365700 0.87592100  B 0.94636800 -2.50212300 0.90486700  B 0.94642000 -2.50307400 -0.90208600  B 0.45797700 2.50689300 -1.36738400  B -2.21127300 -0.36619500 2.74753700  B 2.19972500 -1.99995000 0.00119100  B -1.31976400 2.53161600 1.40088500  B 1.28852400 2.84164800 -0.00135700  B 1.32564800 -0.40266800 2.78622000  B 0.45784500 2.50833700 1.36491900  Ni 2.92905500 -0.03053200 0.00009400  H 4.37271100 0.47074000 -0.00287700 | 129.93 | -2502.531547 | -2502.349944 |
| 11. | H-Cu@B_40_  B -2.32327100 0.34124200 1.82857200  B 0.53554700 -2.83377700 1.69524600  B -1.87233800 -1.23927500 1.42747100  B 3.23269600 0.43531300 -1.76923400  B 2.13683100 2.87251600 -0.00040700  B 0.44448700 1.96758500 2.62021100  B 1.41307600 -1.66697200 -2.38889000  B -0.94678500 1.16467900 -2.34941900  B -2.32308100 0.34059300 -1.82863000  B 0.53564700 -2.83440100 -1.69437300  B -1.87208200 -1.23980300 -1.42725700  B 2.26196400 -0.35689800 2.74367500  B 2.89243400 -1.15630000 1.38788700  B 2.89242800 -1.15673200 -1.38743700  B 2.85034400 1.71282300 -0.88293600  B -1.91920600 1.62341800 0.87362400  B -0.35049100 -1.66061300 -2.38601300  B 2.85038100 1.71315100 0.88250600  B 3.16868700 -1.93652200 0.00034700  B -1.28364400 -0.41435200 2.77022200  B -0.35062000 -1.65986400 2.38655900  B 1.41299700 -1.66616000 2.38941200  B 1.85442000 1.21768100 -2.37831100  B -0.94690300 1.16535800 2.34899100  B 1.85436300 1.21838300 2.37799500  B 1.91928300 -2.49493200 -0.88765600  B 1.91920600 -2.49458400 0.88846800  B 3.23267700 0.43589000 1.76920800  B 1.34297600 2.53437200 1.38969400  B 0.44459500 1.96689000 -2.62082400  B -1.91894000 1.62308500 -0.87419900  B -0.85986800 -2.51681800 -0.89425300  B -0.85992100 -2.51641300 0.89499800  B -0.43843600 2.50863000 1.37624500  B 2.26199700 -0.35775500 -2.74347900  B -2.11789900 -2.00826300 0.00023300  B 1.34307200 2.53403100 -1.39047900  B -1.26200700 2.82851500 -0.00044000  B -1.28348000 -0.41518600 -2.77016700  B -0.43840100 2.50828400 -1.37702800  Cu -3.18753000 -0.01125700 0.00002900  H -4.72528900 -0.10765200 -0.00150800 | 97.37 | -2634.668041 | -2634.486314 |
| 12. | H-Zn@B_40_  B 0.21209600 -1.73182000 -2.71089800  B 3.49606000 -1.70418700 -0.00353000  B 1.81593700 -1.39415500 -2.36627200  B 0.22061400 1.73230700 2.71225900  B -2.30400100 0.00119500 1.67238100  B -1.38035700 -2.66664100 0.00248200  B 2.28625300 2.36990700 0.86634700  B -0.61368200 2.38299200 -1.40137000  B 0.21350300 1.73219800 -2.71087700  B 3.49745100 1.70194100 -0.00351900  B 1.81707400 1.39326700 -2.36626900  B 0.99752800 -2.72392600 1.74398800  B 1.82120100 -1.39301700 2.36393700  B 1.82236900 1.39212000 2.36395400  B -1.07766500 0.87227900 2.33842300  B -1.08333600 -0.86989700 -2.33199600  B 2.28344700 2.36969600 -0.87123700  B -1.07835000 -0.87087600 2.33843600  B 2.59739700 -0.00078500 2.62443800  B 0.99380600 -2.72460400 -1.74535700  B 2.28153600 -2.37098200 -0.87125400  B 2.28436300 -2.37126600 0.86630800  B -0.60975400 2.38347800 1.40405600  B -0.61559100 -2.38201400 -1.40141000  B -0.61165400 -2.38254500 1.40403200  B 3.15964200 0.88373600 1.37331400  B 3.15891800 -0.88579400 1.37336400  B 0.21920100 -1.73204400 2.71225100  B -1.92757900 -1.39957400 0.87629000  B -1.37823500 2.66824000 0.00251100  B -1.08262400 0.87134400 -2.33195600  B 3.15798300 0.88363000 -1.37984100  B 3.15730000 -0.88564800 -1.37989100  B -1.92825300 -1.39760900 -0.86791000  B 0.99969300 2.72363800 1.74400100  B 2.59083500 -0.00076400 -2.62814000  B -1.92656500 1.40167300 0.87629400  B -2.31172200 0.00120800 -1.66393100  B 0.99599500 2.72436300 -1.74532600  B -1.92718000 1.39968000 -0.86786600  Zn -3.85729200 -0.00010900 -0.00238100  H -5.39950900 -0.00045600 0.02035700 | 52.99 | -2773.454282 | -2773.275537 |
